# Test delay and change awareness moderate retroactive and proactive memory effects

**DOI:** 10.3758/s13421-026-01903-4

**Published:** 2026-06-18

**Authors:** Samuel W. Cox, Colleen M. Kelley, James W. Antony, Christopher N. Wahlheim

**Affiliations:** 1 Department of Psychology, University of North Carolina at Greensboro, 559 Eberhart Building, PO Box 26170, Greensboro, NC 27402 USA; 2 Department of Psychology, Florida State University, Tallahassee, FL USA; 3 Department of Psychology and Child Development, California Polytechnic State University, San Luis Obispo, CA USA

**Keywords:** Study-phase retrieval, Reminding, Interference, Facilitation, Integration

## Abstract

Similar experiences can enhance or impair existing memories or newly encoded events. Such retroactive and proactive memory effects have been examined using A-B, A-D tasks in which pairs studied across two lists share cues but have changed responses. These tasks often show greater retroactive interference on immediate than delayed tests and greater proactive interference on delayed than immediate tests. Four experiments using A-B, A-D tasks characterized the role of change awareness in such effects. Retroactive interference from List 2 onto List 1 recall was observed on immediate but not delayed tests. When List 2 included measures of change awareness and List 1 recall, retroactive facilitation in List 1 recall was observed on a delayed test. Conversely, intrusions from List 1 onto List 2 recall, indicating proactive interference, were greater on delayed than immediate tests. Response interdependence on the final test showed that correct recall was associated with recollection of other list responses and that changes had occurred. These findings indicate that retrieval practice during List 2 both increased accessibility of earlier responses and promoted cross-episode integration. When the retrieved response was later the target, this accessibility produced retroactive facilitation; when it was the competitor, it increased proactive interference unless recollection of change preserved information about the responses’ temporal relationship. The theoretical implications are that temporal changes in retroactive and proactive interference depend not only on differences in trace accessibility across lists but also on whether study-phase retrieval promotes integrative encoding and whether change information remains accessible at test.

## Introduction

In daily life, we are tasked with remembering specific details from episodes that have overlapping elements with other episodes. For example, we may need to remember a conversation with a long-term collaborator or where we parked in the usual parking deck. Routine activities like these share many features, and this similarity can impair the successful retrieval of information from a target event. This interference can occur when recent events impair memory for earlier events (retroactive interference) or when earlier events impair memory for recent events (proactive interference). Both forms of interference have long been considered primary determinants of forgetting (for a review, see MacLeod, 2024). One factor that consistently determines the degree of interference is the time that elapses between the most recent learning episode and the memory test. Retroactive interference is greater on immediate tests, whereas proactive interference is greater on delayed tests (Briggs, 1954; Greenberg & Underwood, 1950). Extant theoretical accounts have proposed various strength- and context-based mechanisms to account for these variations in interference, which we summarize below. In the present study, we examined interference differences across delays and focused on the roles of noticing changes between similar events and later recollecting that such changes had occurred.

Awareness of changes during study and test phases has been associated with interference reduction (for reviews, see Wahlheim et al., 2021; Wahlheim & Zacks, 2025), and this has been observed for both proactive and retroactive effects of memory to varying degrees (e.g., Jacoby et al., 2015). Given that forgetting occurs over time, it is reasonable to assume that change awareness and any associated improvements in recall of specific event details will diminish with increasing test delays. However, it is unclear how this assumed reduction in the memory benefits associated with change awareness occurs simultaneously with the opposite effects of test delay on proactive and retroactive interference. To investigate this issue, we report here four experiments conducted by Colleen Kelley, with input from Larry Jacoby, using variants of paired associate (A-B, A-D) interference tasks that included various measures of change awareness.

### Interference effects and underlying mechanisms

We begin with an overview of select literature on interference effects and mechanisms. Because this literature is vast, we focus mostly on studies using paired-associate cued recall. In those paradigms, participants study two lists of cue-response pairs. A-B, A-D (changed) pairs are included to examine the effects of competing associations on subsequent memory. For those pairs, cues (e.g., knee) remain constant across lists, whereas responses change from List 1 (e.g., bone) to List 2 (e.g., bend). To measure interference, the task includes A-B, C-D (control) pairs with different cues and responses in Lists 1 and 2 to assess retroactive and proactive memory effects, respectively. On a subsequent test, participants receive cues and attempt to recall responses from List 1 (to assess retroactive interference) or List 2 (to assess proactive interference). Interference is observed when recall is poorer for changed than control pairs. It is also observed when opposite-list intrusions are emitted for A-B, A-D pairs.

As mentioned above, the degree of retroactive and proactive interference depends on test delays. This was shown early on by Underwood (1948) and Briggs (1954) who used A-B, A-D paradigms to examine how, in the face of retroactive interference from List 2 pairs, the retrieval of List 1 pairs varies between delayed (48 h or 24 h) and immediate tests. Both studies found diminished retroactive interference effects over time. Investigating this more granularly, Koppenaal (1963) used a similar paradigm to assess memory for List 1 responses across seven retention intervals ranging from 1 min to 1 week. Delays shorter than 24 h led to poorer recall from List 1 than List 2, indicating the presence of retroactive interference. Conversely, delays 24 h or longer eliminated the difference between recall from Lists 1 and 2, showing that retroactive interference decreased over time when retention intervals were sufficiently long. The finding of comparable retroactive interference at relatively brief retention intervals extends to situations meant to induce proactive interference. For example, Underwood (1949) found no difference in proactive interference between test delays of 20 and 75 min in an A-B, A-D task. However, subsequent work showed that proactive interference increased from a retention interval of 10 min to intervals of 5 and 48 h (Greenberg & Underwood, 1950) and from 1 min to 1 week (Koppenaal, 1963). Collectively, these findings show that retroactive interference decreases while proactive interference increases with increasing test delays, provided that there are sufficiently long delays.

A robust theory of interference needs to account for the presence of retroactive and proactive interference as well as their differences across test delays. Several theoretical accounts of forgetting and interference have been proposed. Foundational research on forgetting showed that nonsense syllables were forgotten logarithmically as a function of time and attributed the losses of memory traces to decay during the passage of time (Ebbinghaus, 1948). However, an association between time and forgetting does not necessitate that the former is the sole cause of the latter. Indeed, work examining the role of non-temporal factors in forgetting found evidence disconfirming trace decay theory (McGeoch & McDonald, 1931). Participants learned a list of adjectives to one perfect recall, engaged in an interpolated task, and then completed a test. The interpolated phase consisted either of rest or of learning new materials varying in similarity to the original list. Across those conditions, the length of the interpolated phase was constant. Regardless, more similarity in the contents of the original and interpolated phases increased forgetting of the first list, which suggested a role for interference in forgetting.

The response-competition hypothesis was proposed to explain such interference effects (McGeoch, 1932). The account posited that under conditions which promote associative interference (e.g., A-B, A-D tasks), target and non-target information compete for retrieval, leading participants to report the response more strongly associated with the cue. This leads to the prediction that incorrect recall attributed to interference should primarily consist of overt intrusions (i.e., reporting D when the target is B, or vice versa). This claim was tested by evaluating the relative contribution of overt intrusions to an estimate of total retroactive interference when the interpolated phase varied in its potential to create interference (Melton & Irwin, 1940). Overt intrusions constituted a small proportion of total retroactive interference and decreased with more interpolated learning. This suggested that overt intrusions contributed to interference, but unlearning of targets also played a role. This two-factor model influenced later work for decades, with studies reporting evidence for unlearning (Barnes & Underwood, 1959) and additional mechanisms (e.g., response-set interference, Postman & Stark, 1969).

Of relevance to the present study, Baddeley (1976) proposed a trace-decay theory accounting for the differential impact of test delay on retroactive and proactive interference. The theory assumed that the trace strength of events in two studied lists decay exponentially over time. Consequently, List 2 traces should be stronger than List 1 traces at relatively early testing periods. Therefore, if targets are weaker List 1 responses, maximal retroactive interference should occur, whereas if targets are stronger List 2 responses, minimal proactive interference should occur. As test delays increase, the forgetting curves for each list converge and asymptote, resulting in reduction in the trace strength difference responsible for retroactive interference, thus diminishing its effects. List 1 traces also become closer in strength to their List 2 counterparts, thus increasing proactive interference. With few assumptions, this theory accounts for temporally variable interference effects, but an account of interference based solely on trace decay may not be sufficient to completely explain such dynamics.

Further theoretical developments leveraged computational models to test the idea that contextual associations played a critical role in interference and forgetting phenomena. One such model incorporated a contextual fluctuation component into the Search of Associative Memory model (Mensink & Raaijmakers, 1988). The main assumption was that information is stored in memory along with contextual cues that drift gradually over time. At retrieval, multiple contextual cues are activated, and successful retrieval requires a match between encoding and retrieval contexts. This model accounted for the qualitative pattern showing the aforementioned temporal dynamics of proactive interference (Koppenaal, 1963). Although the model was not applied to findings showing decreased retroactive interference at increased test delays, their contextual drift view implies that it should. Specifically, as longer test delays exacerbate the difference between List 2 and retrieval contexts, the effect of interference imposed upon List 1 items by contextual cues associated with List 2 items should diminish. Thus, this model explains changes in interference over time as reflecting decreases in the strength of context signals.

### Change awareness: A framework for retroactive and proactive memory effects

Though no single model can completely explain interference effects, the aforementioned efforts provide a foundation for theoretical development. Here, we acknowledge the constructs of trace strength and contextual processing, and extend theorizing to consider how conscious awareness of the relationship between perceptions and memories contributes to retroactive and proactive memory effects over time. Although the interference literature above focused on mechanisms of impairment, studies from that era also showed that similar events can facilitate memory. This was shown in retroactive memory effects in a foundational study using a variant of the A-B, A-D paradigm in which responses that were semantically similar led to higher recall of List 1 responses as compared to a control condition (Barnes & Underwood, 1959). In contrast, retroactive interference was observed when responses were less similar. Retroactive facilitation was accounted for by a mediation mechanism that associated all elements (A-B-D), and most participants reported using this strategy. Later studies showing such facilitation, but without semantically similar responses, suggested that knowing that responses would change can also lead to facilitation by guiding retrievals of A-B pairs during A-D study trials (e.g., Bruce & Weaver, 1973; Robbins & Bray, 1974). In both studies, facilitation occurred at longer retention intervals and shorter lags between A-B and A-D pairs. The shorter lags likely aided A-D pairs in reminding participants of A-B pairs (Benjamin & Ross, 2010), thus increasing change awareness (also see, Jacoby, 1974). Collectively, these findings suggest that change awareness and the attendant encoding consequences of study-phase retrievals can promote retroactive facilitation.

Larry Jacoby and colleagues pioneered a research program examining the role of change awareness during perception and later remembering in retroactive and proactive memory effects (Jacoby & Wahlheim, 2013; Jacoby et al., 2015; Wahlheim & Jacoby, 2013). They proposed the memory-for-change (MFC) framework, which extends on the recursive remindings hypothesis (Hintzman, 2011) by positing that stimulus features trigger remindings of existing memories, enabling associative encoding of similar events with their temporal relationship (see also Tzeng & Cotton, 1980; Winograd & Soloway, 1985). Accordingly, memory is supported when stimuli and their remindings can be later recollected, as such recursion provides access to metadata about how the study-phase retrieval occurred. Supporting evidence was found in a study examining proactive memory effects in A-B, A-D tasks, showing that awareness of changes both during A-D study and on a later cued-recall test was associated with proactive facilitation, whereas awareness of changes during study but not at test was associated with proactive interference (Wahlheim & Jacoby, 2013). Following that study, Jacoby et al. (2015) showed that instructing participants to engage in study-phase retrievals promoted awareness of changes and led to retroactive and proactive facilitation. Additional studies of retroactive memory effects showed increased remindings of A-B pairs and downstream retroactive facilitation resulting from longer A-D study times (Garlitch & Wahlheim, 2020a; Negley et al., 2018) and interpolated testing of A-B pairs with feedback (Wahlheim et al., 2023). Moreover, these findings are generalizable: similar patterns have been shown in naturalistic contexts such as retroactive misinformation effects in eyewitness memory paradigms (Putnam et al., 2017), proactive effects of exposure to headline content in false news corrections paradigms (Wahlheim et al., 2020), and proactive effects of observed actions in event comprehension paradigms (Wahlheim & Zacks, 2019).

As ongoing work seeks to further specify the cognitive mechanisms which promote change detection and recollection, a critical next step is to evaluate retroactive and proactive memory effects across variable retention intervals. Additionally, the effects of requiring overt reporting of change awareness during study have not been directly compared for retroactive and proactive task designs. Further, paired associate learning tasks using word pairs that offer high experimental control for examining these effects and assessing change awareness have mostly focused on the mnemonic consequences of encoding manipulations on immediate tests (but see Wahlheim et al., 2026). Therefore, the role of change awareness in the dynamics of retroactive and proactive memory effects over time remains unclear. In the present study, four experiments attempt to fill this knowledge gap by directly comparing retroactive and proactive memory effects on immediate and delayed tests in tasks that also assessed awareness of changes.

### The present study

**Fig. 1:**
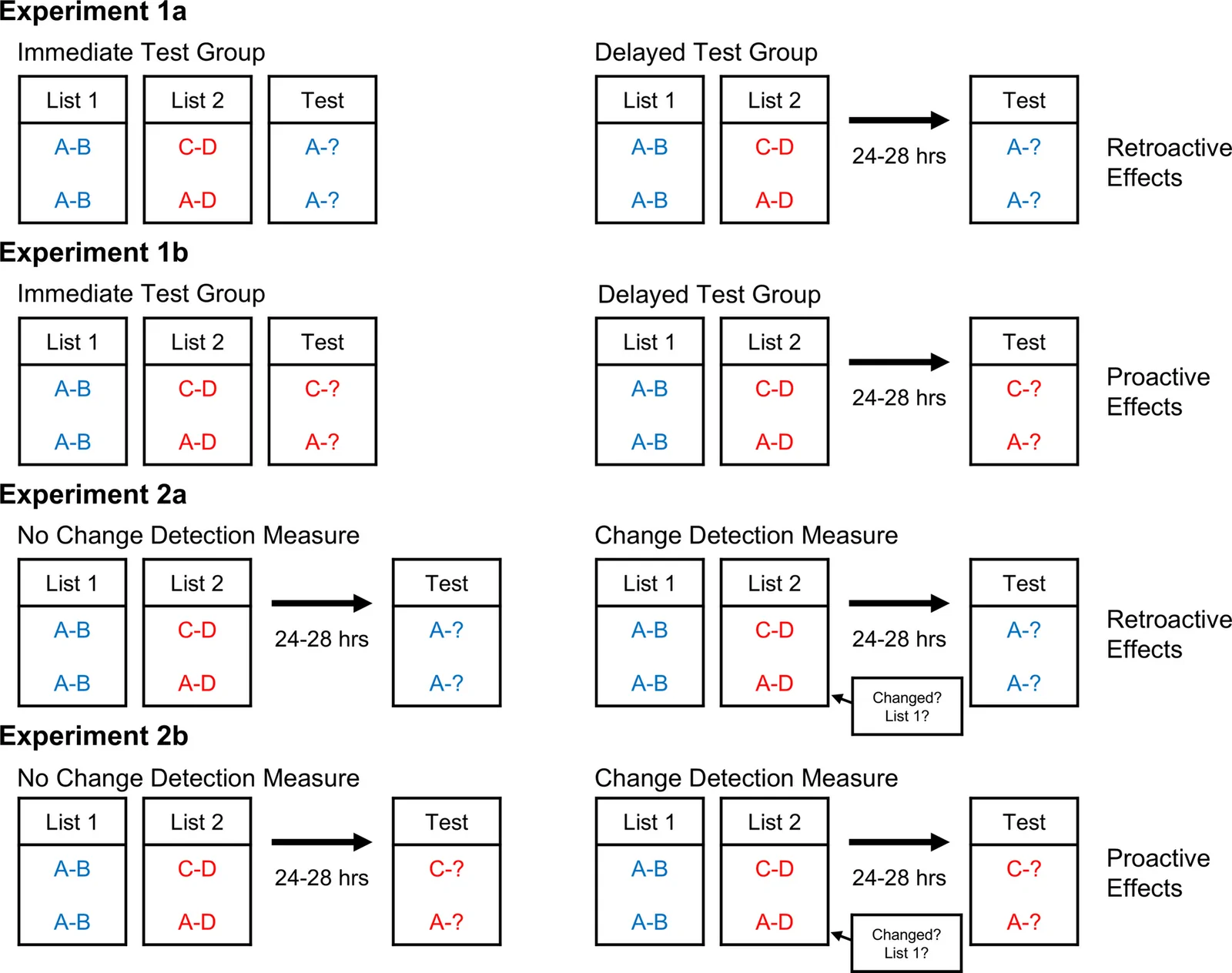
Illustration of experimental groups. The groups in both experiments varied on whether the final test required cued recall from List 1 or List 2 first on each test trial. Retroactive effects of memory were assessed by testing List 1 responses first. Proactive effects of memory were assessed by testing List 2 responses first. The groups in Experiment 1 also varied on whether the final test appeared immediately after List 2 or in a second session that started after a 24- to 28-h delay. The groups in Experiment 2 both completed delayed tests and also varied on whether change detection and List 1 recall was overtly measured. The item type conditions were manipulated within-subject and are represented symbolically above based on the relationship between cues and responses in Lists 1 and 2. Control items included pairs without shared elements across lists (A-B, C-D) and served as the contrast for assessing overall retroactive and proactive effects of memory. Changed items included pairs with shared cues and different responses across lists (A-B, A-D)

Our approach here builds on studies using A-B, A-D variants with word-pair stimuli and cued-recall tests that assess memory for both responses and whether they changed across lists. Figure 1 displays a schematic of the experiments. Participants first studied two lists of cue-response word pairs sequentially because associations between items are better established when they are encoded in the same session (Zeithamova & Preston, 2017). The lists included unique control pairs (A-B, C-D) and pairs with cues that repeated and responses that changed across lists (A-B, A-D). Participants then took a final cued-recall test that required them to recall the response from List 1 (B) or List 2 (D), indicate if the response with that cue had changed across lists, and if so, recall the response from the opposite list, List 2 (D) or List 1 (B). Based on prior findings (Jacoby & Wahlheim, 2013), we assumed that when participants remembered changes and recalled the non-target response at test, this nearly always indicated that participants were aware of changes in List 2 and engaged in study-phase retrievals of List 1 pairs.

Within experiments, we used between-subjects manipulations to compare the effects of test delay (Experiments 1a and 1b) and overtly indicating change awareness and recall of List 1 responses during List 2 study (Experiments 2a and 2b). Experiments 1a and 1b identified differences in retroactive and proactive memory effects immediately and after 1 day without an overt measure in List 2. Experiments 2a and 2b examined overt change awareness and study-phase retrieval effects by requiring half of the groups to identify changed pairs and recall List 1 responses during List 2 study; these groups were tested after 1 day to increase sensitivity to the subsequent memory effects of retrieving from List 1 during List 2 because retrieval-enhanced learning is often greater after a delay (for a review and meta-analysis, see Rowland, 2014).

**Fig. 2:**
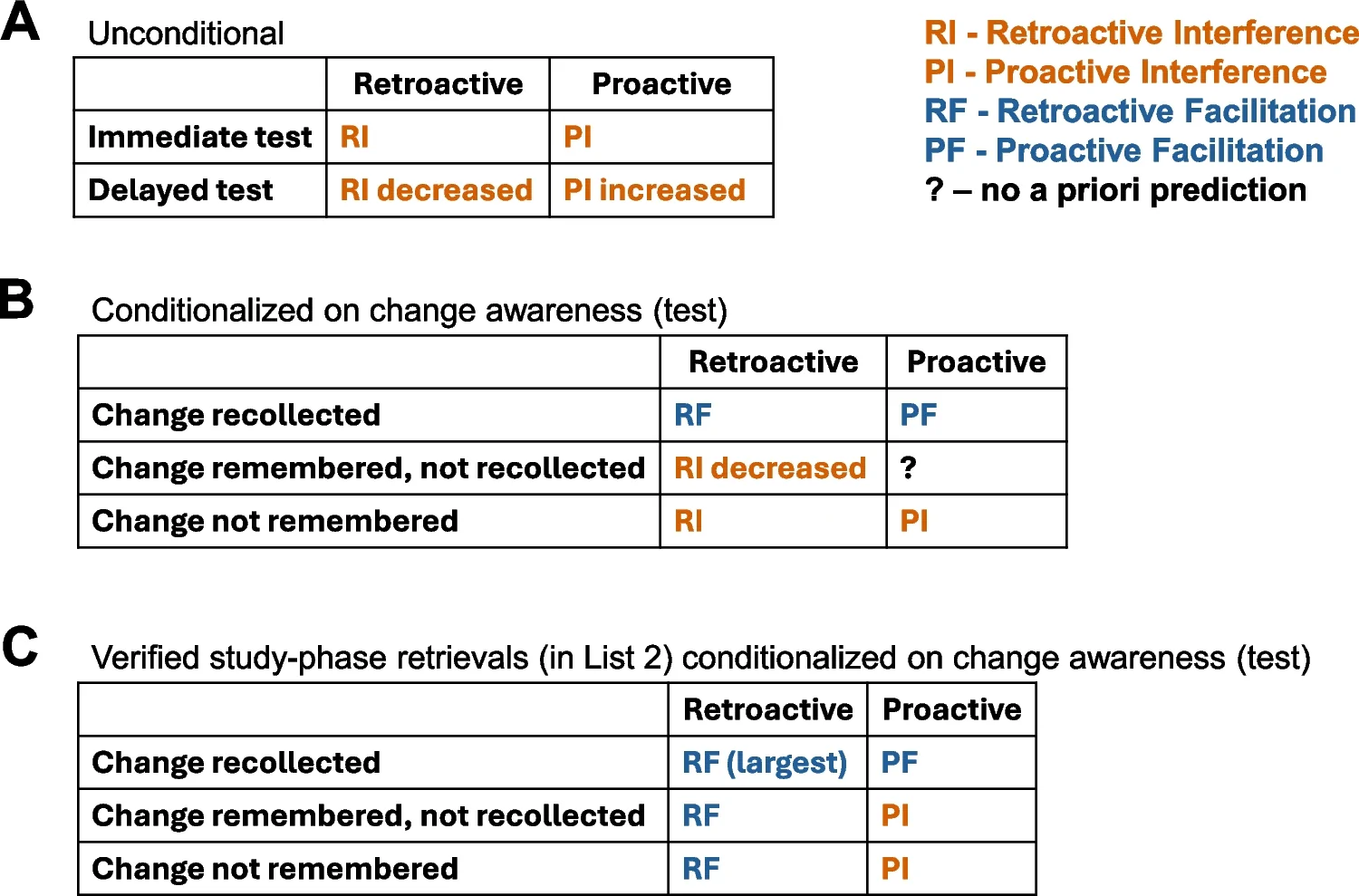
A priori predictions for retroactive and proactive memory effects across levels of analysis. Panel A shows predictions for unconditional analyses: retroactive interference (RI) on the immediate test decreasing on the delayed test, and proactive interference (PI) on the immediate test increasing on the delayed test. Panel B shows predictions conditionalized on change awareness at test: facilitation when changes are recollected, reduced RI and no prediction for the intermediate change awareness, and interference when changes are not remembered. Panel C shows predictions for recall following verified study-phase retrievals in List 2 (and detected changes) conditionalized on change awareness at test: retroactive facilitation (RF) across all change-awareness states (largest when changes are recollected), and proactive facilitation (PF) only when changes are recollected. Note that the direction and magnitude of effects in Panels B and C are expected to vary depending on factors such as test delay, whether change detection is measured in List 2, and stimulus characteristics (see the text for more details)

We assessed interference and facilitation effects by comparing correct recall from target lists for A-B, C-D and A-B, A-D pairs. We also assessed the interference effects by comparing intrusions from non-target lists for A-B, A-D pairs across the test delay and List 2 measures groups. Interference was shown when correct recall was lower for A-B, A-D than A-B, C-D pairs and/or when intrusions were observed. Conversely, facilitation was shown when correct recall was higher for A-B, A-D than A-B, C-D pairs. To anticipate the patterns of results here, it is important to consider differences in how change awareness and study-phase retrievals are presumed to benefit memory accuracy (see also Jacoby et al., 2015). For retroactive effects, study-phase retrievals occur for target responses, thus leading to facilitation driven by retrieval practice and to a lesser degree by associative encoding with List 2 responses. Conversely, for proactive effects, study-phase retrievals occur for non-target responses, which increases the accessibility of competitors. However, this retrieval-enhanced accessibility of List 1 responses also aids successful memory for List 2 responses to the extent that both responses are associatively (recursively) encoded. This places a heavier demand on subsequent recollection of contextual details, such as metadata about how List 2 items evoked retrievals of List 1 items, thus marking their temporal contexts (i.e., list memberships) by imbuing the items with attributes of change. Figure 2A summarizes a priori predictions for unconditional recall across test delays and memory types; Fig. 2B and C summarize predictions for the two types of conditional analyses, which we introduce in the relevant *Results* subsections below.

For Experiments 1a and 1b, findings from paired associate learning studies led us to expect more retroactive interference on immediate than delayed tests (e.g., Antony et al., 2022; Antony & Bennion, 2023; Briggs, 1954; Chandler, 1993) and more proactive interference on delayed than immediate tests (Greenberg & Underwood, 1950). In Experiments 2a and 2b, findings from the retrieval practice (for a review and meta-analysis, see Rowland, 2014) and memory for change (Jacoby et al., 2015) literatures led us to expect retroactive facilitation after overt indications of change awareness and study-phase retrievals during List 2. We did not have a priori expectations about the consequences of such overt responses for proactive effects. Increased List 1 accessibility increases the potential for responses from both lists to become associated and thus facilitate memory. However, it also increases the potential for List 1 responses to interfere because of their heightened accessibility. Thus, the balance of these effects, which should depend on how change attributes are preserved until test, should determine the overall pattern of recall. More generally, we expected that memory for changes expressed at test and recall conditioned on those and List 2 responses regarding changes would reflect differences created by the manipulations above. Given the complexity of the recall dependencies that arise in conditional analyses of this sort, we defer more detailed descriptions of hypotheses about the conditional results until just before presenting those analyses below.

## General methods

### Participants

We present the methods for all experiments together because they are highly similar. Participants were recruited online via Amazon Mechanical Turk (AMT) and Qualtrics. They were compensated $3.00 for 1 h of participation across two online sessions. The final sample (after exclusions) comprised 320 participants (80 per experiment; 40 per group).

Experiments 1a and 1b were conducted simultaneously and included 102 women and 58 men aged 18–56 years (*M* = 30.8, *SD* = 8.9 years). Experiments 2a and 2b were conducted simultaneously and included 98 women and 62 men aged 18–58 years (*M* = 30.1, *SD* = 7.8 years). We excluded an additional 75 participants in Experiments 1a and 1b, and 66 participants in Experiments 2a and 2b for not completing the second session on time (Experiments 1a and 1b = 53; Experiments 2a and 2b = 46), writing down items during study (Experiments 1a and 1b = 10, Experiments 2a and 2b = 17), failing the attention check (Experiments 1a and 1b = 1, Experiments 2a and 2b = 0), and for miscellaneous technical issues or failing to follow the instructions (Experiments 1a and 1b = 11, Experiments 2a and 2b = 3).

### Designs

At the outset of both experiments, participants were told that they would study two lists of word pairs and would then attempt to recall words from one of the lists. All participants received a within-subject manipulation of item type including Control (A-B, C-D) pairs with elements that only appeared in one list and Changed (A-B, A-D) pairs with cues that appeared in both lists with changed responses. In each experiment, we also assigned participants to one of two between-subjects groups (see Fig. 1). Experiment 1a examined retroactive effects of exposure to List 2 on recall from List 1. Experiment 1b examined proactive effects of exposure to List 1 on recall from List 2. Both of those experiments examined whether retroactive and proactive effects varied across delays by administering a cued-recall test immediately after List 2 in the same session or after a 24- to 28-h delay in a second session. Experiments 2a and 2b examined retroactive (Experiment 2a) and proactive (Experiment 2b) memory effects on only delayed tests that were administered after a 24- to 28-h delay in a second session. Those experiments also examined the effects of change awareness and study-phase retrieval (i.e., List 1 recall during List 2) by varying the presence of such overt measures on List 2 study trials.

### Procedures and materials

Consented participants were informed that the experiment comprised two sessions and that the second session would begin 24 h after Session 1. During Session 1, participants studied 32 word pairs in each of two lists. Pairs included cue-response semantic associates taken from (Eakin, 2005). Sixteen control pairs appeared only in the list that would later be tested (e.g., “MEAL-LUNCH,” “FEET-SHOES”), and 16 changed pairs appeared with the same cue and different responses in each list (e.g., “FRUIT-JUICE,” “FRUIT-APPLE”). Pairs appeared for 7 s each in both lists individually and in random order. To ensure that performance was in a sensitive portion of the scale, participants were told to visualize pairs to prepare for a memory test and rate the vividness of each mental image after a pair disappeared. Participants in the Experiments 2a and 2b groups that required overt judgments of change during List 2 pressed a button to indicate whether the same cue appeared with a different response than in List 1. When participants indicated that this was the case, they were prompted to type in the List 1 response. All participants completed an attention check control question after List 1.

After Session 1, participants were told that they would receive an email in 24 h with a link that they must click within 4 h to complete Session 2. Both immediate and delayed test groups were required to return for Session 2 to equate attrition rates. Regardless of when the cued-recall test was administered, participants were asked if they wrote down any pairs while studying and if they used any study strategies other than imagery to affect their cued-recall performance. These questions provided another basis for excluding participants who did not follow instructions.

Participants then received a cued-recall test for either List 1 (retroactive effects) or List 2 (proactive effects). Participants in the immediate test groups received the test right after List 2, whereas participants in the delayed test groups received the test in the second session 24–28 h after List 2, depending on when they accessed the link. Test cues appeared individually and in random order. For each cue, participants were prompted to recall the response from the target list, indicate if responses changed between lists using the scale (“It may have changed,” “It likely changed,” or “I remember that it changed”), then report the response from the non-target list. For consistency with prior studies, we dichotomized this response scale by counting the most confident response as remembering change and the other responses as not remembering change.

### Statistical methods

We performed all analyses using R software, version 4.5.2 (R Core Team, 2025) and functions from the following packages: *tidyverse*, version 2.0.0 (Wickham et al., 2019), *magrittr*, version 2.0.4 (Bache & Wickham, 2025), *patchwork*, version 1.3.2 (Pedersen, 2025), and *sandwich*, version 3.1.1 (Zeileis, 2004; Zeileis et al., 2020). We analyzed the data using mixed-effects models from the *lme4* package, version 1.1.38 (Bates et al., 2015), with by-subject and by-item random intercept effects as well as fixed effects of experimental conditions and groups. Including by-subject random intercepts accounted for individual differences in overall memory ability and including by-item random intercepts accounted for normative differences in item memorability, together reducing the likelihood that effects reflect these confounds rather than the experimental manipulations of interest. However, we note that this approach does not account for idiosyncratic differences in item memorability that are unique to individual participants. Models with singular fits were simplified by removing random intercepts with near-zero variance; further deviations from our statistical approach were applied where necessary and are described below. The model specifications are available in the scripts on the Open Science Framework (OSF) at: https://osf.io/euqmj/. We performed Wald’s χ^2^ hypothesis tests using the *Anova* function of the *car* package, version 3.1.3 (Fox & Weisberg, 2019) and pairwise comparisons using the *emmeans* function from the *emmeans* package, version 2.0.1 (Lenth & Piaskowski, 2025) with Tukey’s method. We report odds ratios (*OR*s) as effect sizes for pairwise comparisons from logistic mixed-effects models. The significance level was α =.05.

## Results and discussion

We present results separately for each pair of experiments to characterize proactive and retroactive memory effects and the role of change awareness over time (Experiments 1a and 1b) and when change awareness is measured immediately following changes (Experiments 2a and 2b). Within each pair, we first report overall cued-recall performance, followed by analyses of change awareness, and then cued recall conditionalized on measures of change awareness.

### Experiments 1a and 1b

#### Overall cued-recall performance

##### Correct recall from target lists

**Fig. 3:**
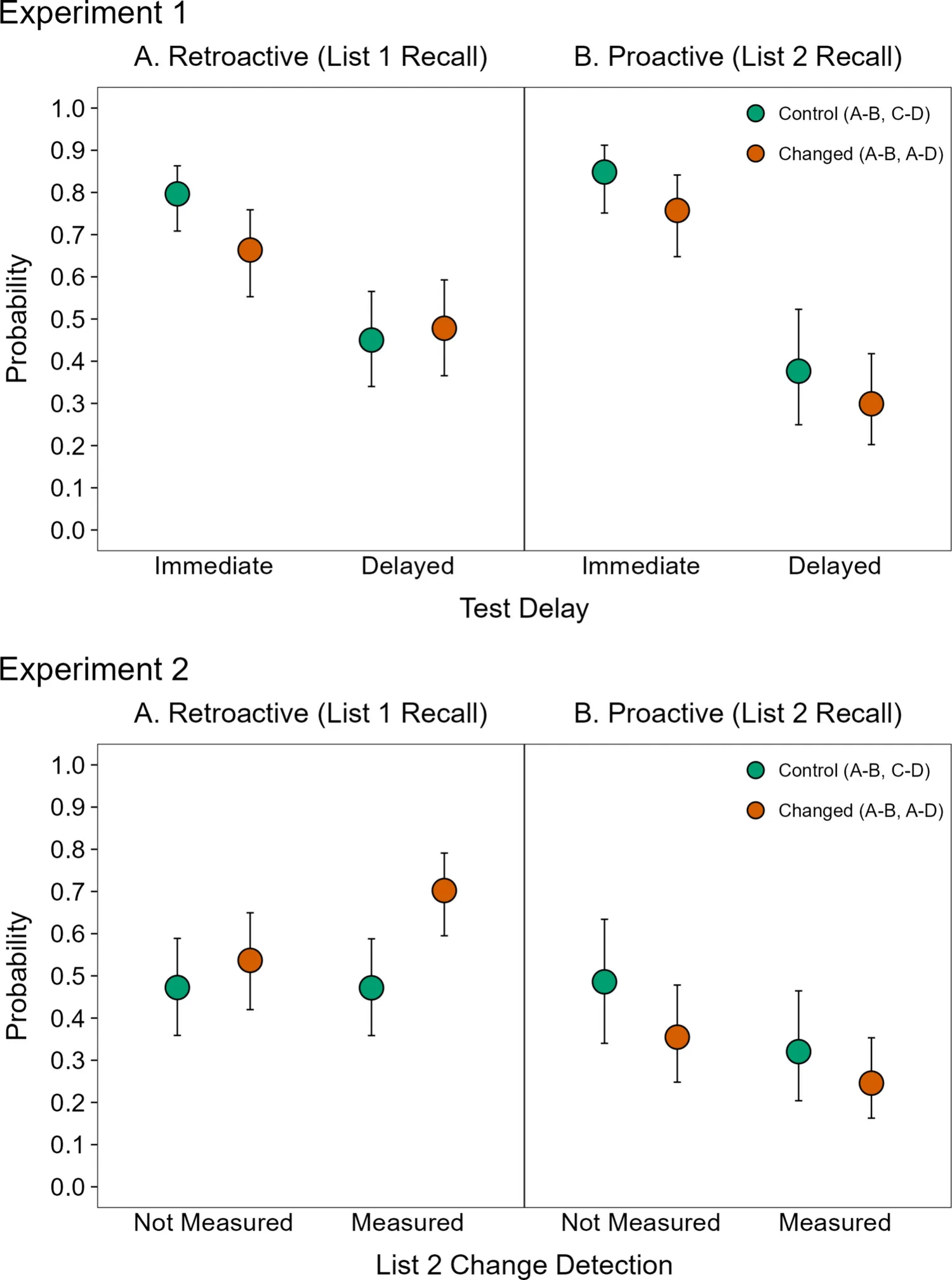
Correct recall from target lists for control and changed items. Points are probability estimates from mixed-effects models with 95% confidence intervals

We first examined retroactive and proactive memory effects by comparing correct recall from target lists in Experiments 1a and 1b (Fig. 3, top panels), respectively. We tested for differences between item types across test delays using separate models for List 1 recall (retroactive effects) and List 2 recall (proactive effects), with fixed effects of Item Type (Control vs. Changed) and Test Delay (Immediate vs. Delayed).

**Retroactive effects**

The model for List 1 recall yielded significant effects of Item Type, χ2 (1) = 6.91, p <.01, OR = 1.33, Test Delay, χ2 (1) = 24.28, p <.001, OR = 3.21, and their interaction, χ2 (1) = 16.93, p <.001. List 1 recall was significantly higher on the immediate than on the delayed test. On the immediate test, List 1 recall was significantly lower for changed than control items, z ratio = 4.81, p <.001, OR = 0.50, showing a retroactive interference effect. However, on the delayed test, List 1 recall was not significantly different between item types, z ratio = 0.86, p =.39, OR = 1.12, showing an elimination of the retroactive interference effect over time.

**Proactive effects**

The model for List 2 recall yielded no significant effect of Item Type, χ^2^ (1) = 2.58, *p* =.11, *OR* = 1.59, a significant effect of Test Delay, χ^2^ (1) = 49.84, *p* <.001, *OR* = 8.23, and no significant interaction, χ^2^ (1) = 1.28, *p* =.26. List 2 recall was significantly higher on the immediate than on the delayed test, but no proactive interference effects were observed.

##### Intrusions from non-target lists

**Fig. 4:**
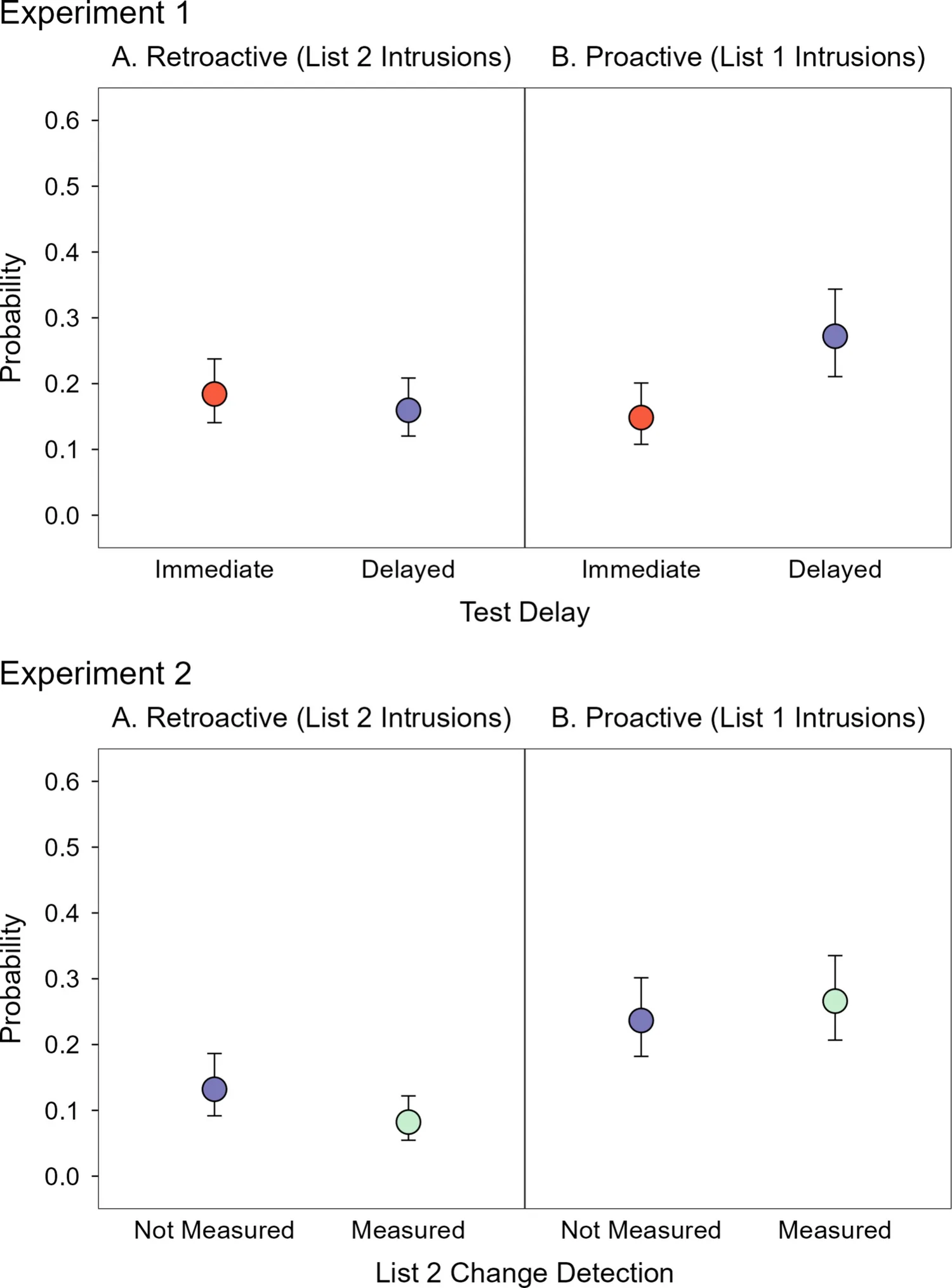
Intrusions from non-target lists for changed items only. Points are probability estimates from mixed-effects models with 95% confidence intervals

We next examined retroactive and proactive memory effects by comparing intrusions from non-target lists for changed items within Experiments 1a and 1b (Fig. 4, top panels), respectively. We tested for differences across test delays using separate models for List 2 intrusions during List 1 recall (retroactive effects) and List 1 intrusions during List 2 recall (proactive effects), with a fixed effect of Test Delay.

**Retroactive effects**

The model for List 2 intrusions yielded no significant effect of Test Delay, χ^2^ (1) = 0.85, *p* =.36, *OR* = 1.19, indicating that this type of retroactive interference effect did not differ over time.

**Proactive effects**

The model of List 1 intrusions yielded a significant effect of Test Delay, χ^2^ (1) = 14.24, *p* <.001, *OR* = 2.15. Intrusion rates were higher on the delayed than the immediate test, indicating that this type of proactive interference effect became larger over time.

##### Summary

Collectively, these results are generally consistent with the existing literature in showing more evidence for retroactive interference on immediate than delayed tests and more evidence for proactive interference on delayed than immediate tests, albeit asymmetrically across measures of correct recall from target lists and intrusions from non-target lists.

### “Changed” classifications at test

We next examined change awareness at the time of test to characterize the observation differences in the conditional analyses of cued recall reported in the next section. Table 1 (left columns) displays the probabilities of the three “changed” classifications at test. *Changed + List 1 or List 2 Recalled* refers to instances when participants classified changed items as such and recalled the non-target list response. *Changed + List 1 or List 2 Not Recalled* refers to instances when participants classified changed items as such but did not recall the non-target list response. *Not Changed + List 1 or List 2 Not Recalled* refers to instances when participants did not classify changed items as such and were thus not prompted to recall non-target list responses. Because all three classifications are exhaustive probabilities that sum to one within each group in each experiment, including all three levels in a single model would introduce perfect linear dependence among predictors. To circumvent this issue, we modeled two classification levels reflecting qualitatively different types of memory for change and treated the third as the reference level, so that effects are estimated as contrasts relative to the omitted level. We specifically modeled the levels implying awareness of changes at test, assuming that such awareness was based more on recollection of the earlier experience of detecting changes when participants could also recall the non-target response at test (Negley et al., 2018; Wahlheim & Zacks, 2019). Because recollection of event details degrades faster than the more general feeling of prior experiences (Reyna & Brainerd, 1995), we expected that participants would exhibit recollection-based awareness of changes more often on the immediate than on the delayed test.

**Table 1:** “Changed” classifications at test for changed items as a function of target list, test delay, and change detection measure

		Experiment 1		Experiment 2	
Target List	Test Classification	Immediate +	Delayed +	Delayed +	Delayed +
(Memory Effects)		No Measure	No Measure	No Measure	Measure
Recall List 1	Changed + List 2 Recalled	.43	.24	.24	.31
(Retroactive)	Changed + List 2 Not Recalled	.21	.28	.28	.31
	Not Changed	.36	.48	.48	.38
Recall List 2	Changed + List 1 Recalled	.47	.24	.26	.34
(Proactive)	Changed + List 1 Not Recalled	.18	.25	.27	.20
	Not Changed	.35	.51	.47	.46

The proportions above correspond to the relative cell sizes underlying the conditional probability estimates shown in Figs. 6 and 7. Dispersion measures are not shown because the values above are reported descriptively to illustrate the distributions of observations contributing to the conditional analyses rather than to estimate population parameters

#### Response probabilities for “changed” classifications

**Fig. 5:**
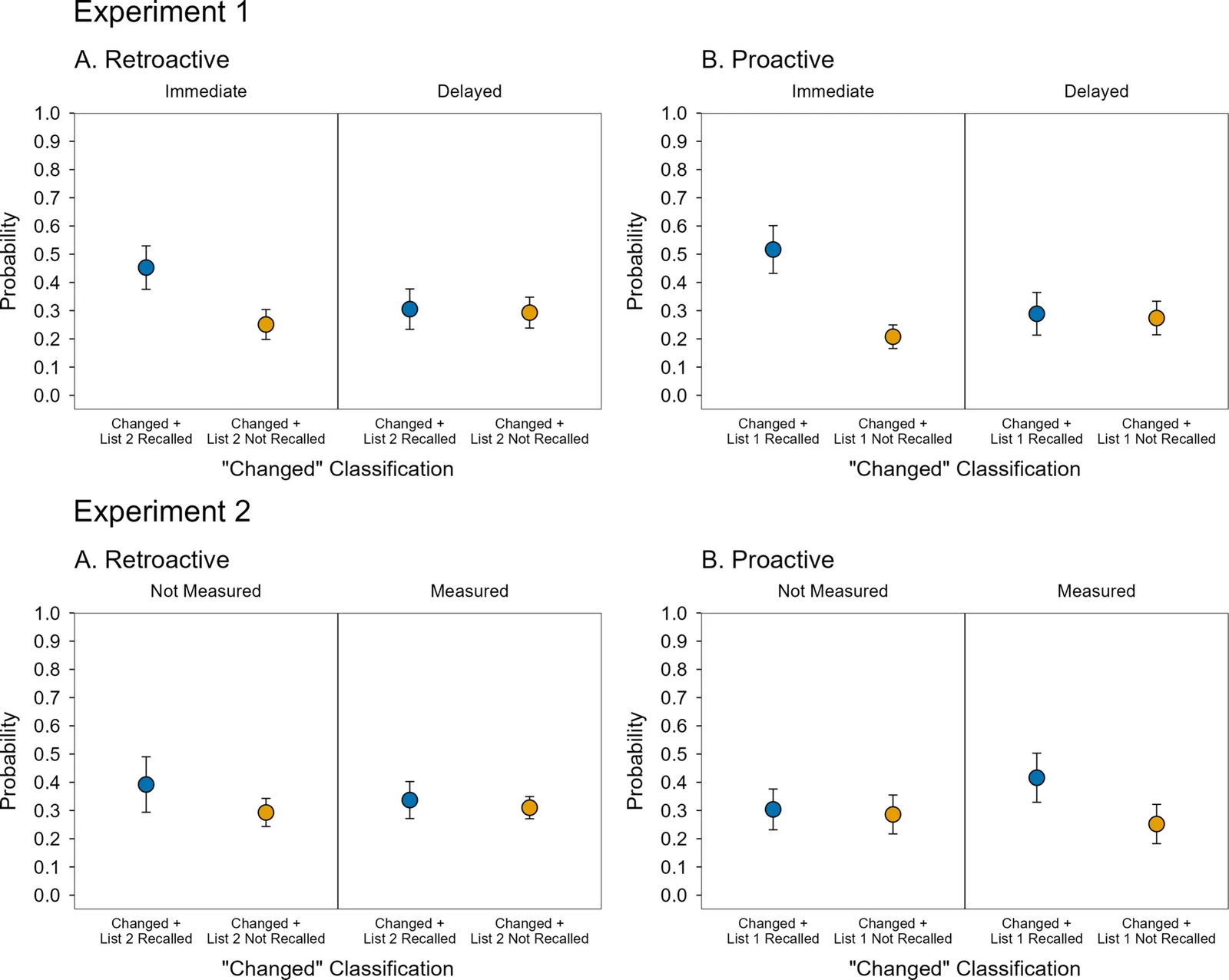
Probabilities of change awareness at test for changed items. Points are probability estimates from regression models with 95% confidence intervals

For both Experiment 1a and 1b, mixed-effects models with fixed effects of Test Classification (Changed + Non-Target Recalled vs. Changed + Non-Target Not Recalled) and Test Delay and by-subject random intercepts exhibited singular fits. Removing the only random intercept eliminated the random-effects structure, so probabilities were compared using two separate multiple regression models with the same predictors. To account for within-subject dependence, subject-clustered robust standard errors were used for hypothesis testing and pairwise comparisons (for a review, see McNeish, 2023). We report Cohen’s *d* effect sizes for pairwise comparisons and partial eta-squared (*η*^*2*^*p*) for omnibus effects from these models. For Experiments 1a and 1b, the model-implied probabilities for each combination of Test Classification and Test Delay are displayed in Fig. 5 (top panels).

**Retroactive effects**

The model for retroactive effects (Experiment 1a) yielded significant effects of Test Classification, *F*(1, 76) = 15.35, *p* <.001, *η*^*2*^*p* =.168, Test Delay, *F*(1, 76) = 7.79, *p* <.01, *η*^*2*^*p* =.093, and their interaction, *F*(1, 76) = 7.58, *p* <.01, *η*^*2*^*p* =.091. Pairwise comparisons within the Test Delay groups showed that, on the immediate test, change awareness was significantly more likely to be accompanied by recall of List 2 responses than to occur without such recall, *t*(76) = 3.92, *p* <.001, *d* = 0.90; on the delayed test, this difference was not significant, *t*(76) = 0.27, *p* =.79, *d* = 0.06. These results suggest that memory for changes relied less on recollection over time.

**Proactive effects**

The model for proactive effects (Experiment 1b) also yielded significant effects of Test Classification, *F*(1, 77) = 38.87, *p* <.001, *η*^*2*^*p* =.335, Test Delay, *F*(1, 77) = 15.97, *p* <.001, *η*^*2*^*p* =.172, and their interaction, *F*(1, 77) = 17.81, *p* <.001, *η*^*2*^*p* =.188. Pairwise comparisons within the Test Delay groups showed that, on the immediate test, change awareness was significantly more likely to be accompanied by recall of List 1 responses than to occur without such recall, *t*(77) = 6.24, *p* <.001, *d* = 1.42; on the delayed test, this difference was not significant, *t*(77) = 0.31, *p* =.76, *d* = 0.07. These results suggest that memory for changes again relied less on recollection over time.

### Correct recall for changed items conditionalized on “changed” classifications at test

In this section, we assess the extent to which differences in the overall retroactive and proactive memory effects for changed items described above reflected associations with awareness of changes measured at test. To do this, we followed earlier approaches to inferring cross-episode binding during study from the extent to which responses on the cued-recall test depended on remembering that responses had changed and subsequent correct recall of responses from non-target lists. Prior studies showed that manipulations and participant-group differences that influence change detection during study comparably affected subsequent remembering that changes occurred and recall of non-target list responses (Fiedler et al., 2026; Wahlheim & Zacks, 2019). However, test measures underestimate awareness of changes during study because such initial awareness is partially lost to forgetting. The following cells indicating memory for changes thus reflect associations with the most memorable change detection experiences.

For analyses of correct recall from target lists, we conditionalized performance on all three levels of “changed” classifications described in the previous section. These analyses also included target-list recall estimates for control items, allowing assessment of the temporal dynamics of interference and facilitation effects moderated by different qualities of memories for change. Figure 2B summarizes a priori predictions for these analyses, reflecting the expected asymmetry between retroactive conditions, where study-phase retrievals increase target-list accessibility, and proactive conditions, where such retrievals increase alternate-list accessibility. Specifically, we expected recollection-based memory for changes to be universally associated with facilitation, shown as target-list recall that is higher for changed than control items, whereas the complete absence of memory for change would be associated with interference, shown as lower target-list recall of changed than control items. The prediction for the intermediate state of memory for change that is less likely to be based on recollection is less straightforward. Such memory for change should still lead to facilitation under retroactive conditions, as this could reflect remnants of having detected earlier changes that often, but not always, were accompanied by study-phase retrievals. Conversely, for proactive effects, prior work has shown heterogeneity in this cell; it varies in ways that may be systematic, but are not yet fully understood (e.g., Wahlheim et al., 2019). We therefore did not have a clear prediction for this cell. For intrusions, which indicate relative differences in another manifestation of interference, we conditionalized performance similarity, but excluded control items, which were unnecessary for inferences about intra-experimental interference. Although recalls from non-targets lists at test often negate intrusions for most trials (i.e., they are the same responses, and people rarely reported them twice), we maintained the tripartite classification scheme for intrusion analyses for completeness.

#### Correct recall from target lists

**Fig. 6:**
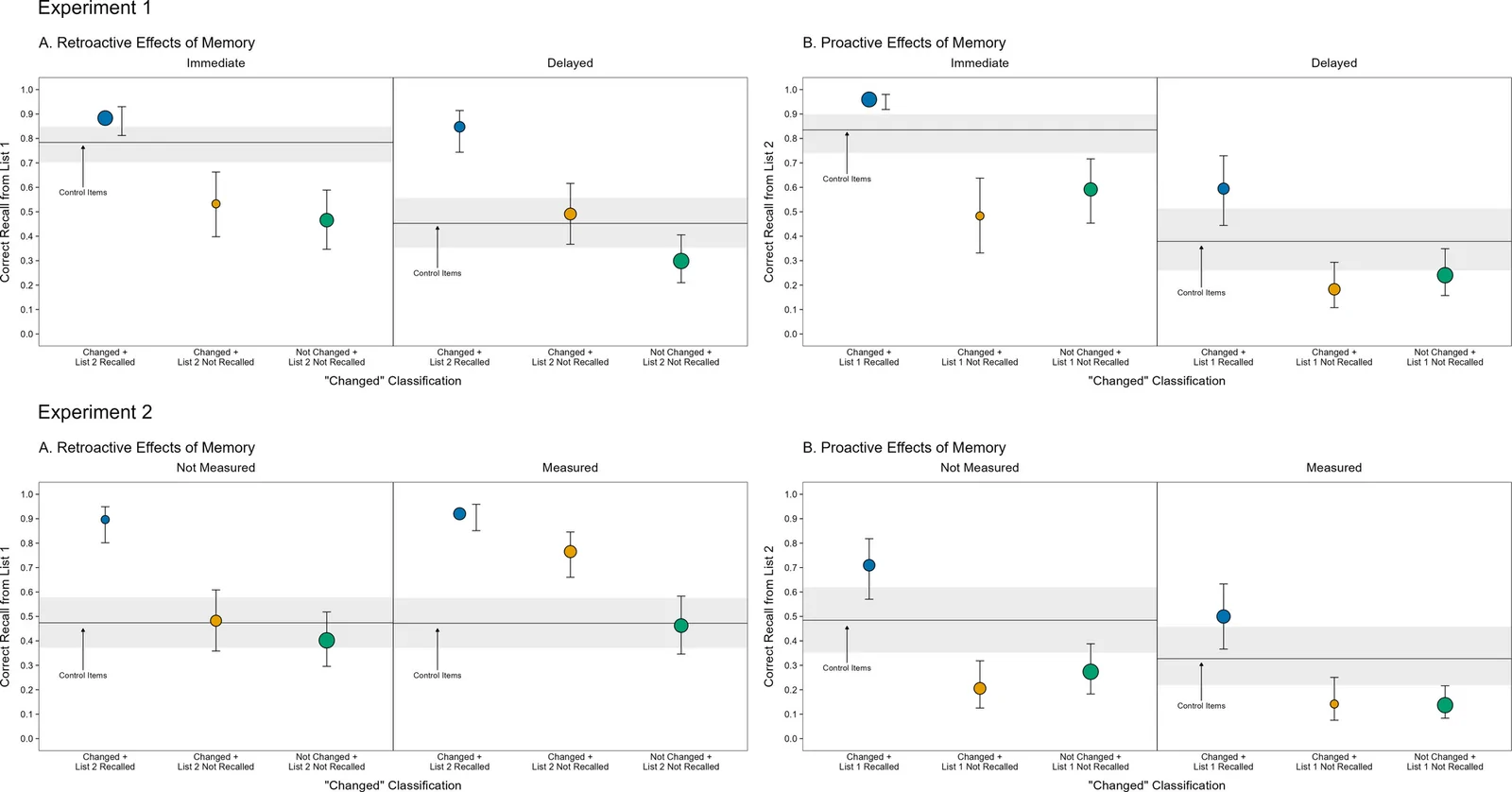
Correct recall for changed items conditionalized on “changed” classifications. Points are probabilities estimated from mixed-effects models. Horizontal bars are model estimates for Control (A-B, C-D) items. Error bars and shaded regions are 95% confidence intervals. Point areas indicate relative differences in the proportions of observations in each cell. Those proportions are displayed in Table 1

We expected to find dependence in recall of responses from both lists shown in conditional probabilities of target list recall being highest when non-target list responses were also recalled for changed items classified as such. We assumed that these instances occurred primarily for items that elicited successful change detection and recall of List 1 responses during List 2 study. The combination of the magnitude of recall differences across classification types and the observation counts for each type determined rates of overall correct recall and non-target list intrusions. Figure 6 (top panels) shows rates of conditional correct recall from target lists for Experiments 1a and 1b. We tested for differences among item type conditions across test delay groups separately for each test type group using separate models for List 1 recall (Fig. 6A) and List 2 recall (Fig. 6B), each including fixed effects of Classification (all three “changed” classifications + control items) and Test Delay. For each test type, we begin by reporting recall differences among the three levels of “changed” classification across test delays before examining the differences between changed and control items. The latter characterize the magnitudes of retroactive and proactive memory effects associated with memory for change, thus indicating their contribution to the overall observed differences in retroactive and proactive memory effects across test delays.

**Retroactive effects**

Prior work using immediate tests has shown that recollection-based memory for change is associated with retroactive facilitation, the absence of memory for change is associated with retroactive interference, and non-recollection-based memory for change is associated with intermediate recall performance (Garlitch & Wahlheim, 2020a; Negley et al., 2018). These findings are consistent with the view that retrieval practice of List 1 responses during change detection and associative encoding of changed responses are associated with better subsequent memory, whereas their absence is associated with greater vulnerability to interference. We expected to replicate those effects here and examined the extent to which their magnitudes differed after a delay, when recollection-based retrieval was less likely to occur.

The List 1 recall model yielded significant effects of Test Delay, χ^2^ (1) = 24.67, *p* <.001, *OR* = 1.95, Classification, χ^2^ (3) = 132.68, *p* <.001, and their interaction, χ^2^ (3) = 28.19, *p* <.001. List 1 recall differed significantly across classification types: it was highest for Changed + List 2 Recalled, intermediate for Changed + List 2 Not Recalled, and lowest for Not Changed + List 2 Not Recalled, with all pairwise differences being significant, smallest *z* ratio = 3.08, *p* <.01, *OR* = 1.72. Pairwise comparisons of changed and control items showed significantly higher recall in the Changed + List 2 Recalled than control cell on the immediate test, *z* ratio = 3.10, *p* =.01, and delayed test, *z* ratio = 6.65, *p* <.001, indicating retroactive facilitation that was larger on the delayed than immediate test (*ORs* = 6.73 vs. 2.09). In contrast, the other conditional cells showed interference on the immediate test, smallest *z* ratio = 5.13, *p* <.001, *OR* = 0.31, but on the delayed test, significant interference remained only for Not Changed + List 2 Not Recalled, *z* ratio = 3.85, *p* <.001, *OR* = 0.51; Changed + List 2 Not Recalled did not differ from control items, *z* ratio = 0.78, *p* =.87, *OR* = 1.17.

Overall, these conditional analyses indicate that the absence of retroactive interference in overall recall after a delay reflected a shift in the mixture and magnitude of underlying effects. Retroactive facilitation was larger on the delayed than on the immediate test when changes were recollected, interference was absent on the delayed test when changes were remembered on non-recollective bases, and interference was present but reduced when changes were not remembered at all. Together, these magnitude changes explain how overall retroactive interference can disappear on a delayed test even as recollection-based memory for changes declines over time.

**Proactive effects**

Compared to retroactive effects, the interplay of interference and facilitation differs under proactive memory conditions because responses retrieved when changes are detected are competitors instead of targets (Jacoby et al., 2015). Detecting change during List 2 often entails recalling the List 1 response, which can leave that response relatively more accessible at test and correspondingly increase proactive interference when change is not later recollected. In contrast, proactive facilitation is expected when changes are recollected, because recollection of change indicates memories in which information about how the two events became associated remains accessible, supporting retrieval of the changed response despite competition from the earlier response. We expect that these different underlying dynamics will still lead to a somewhat similar conditional recall pattern for changed items under proactive memory conditions. However, the potential for facilitation should be lower here, especially after a delay, when List 1 competitors that received retrieval practice have become more accessible and recollection has become less likely to support memory for those items and their list membership.

The List 2 recall model yielded significant effects of Test Delay, χ^2^ (1) = 53.21, *p* <.001, *OR* = 7.11, Classification, χ^2^ (3) = 113.26, *p* <.001, and their interaction, χ^2^ (3) = 12.43, *p* <.01. List 2 recall differed across classification types: it was significantly higher for Changed + List 1 Recalled than both other conditional cells, smallest *z* ratio = 9.37, *p* <.001, *OR* = 8.71, which did not significantly differ from each other, *z* ratio = 1.95, *p* =.21, *OR* = 0.68. Pairwise comparisons of changed and control items showed significantly higher recall in the Changed + List 1 Recalled than control cell on the immediate test, *z* ratio = 3.80, *p* <.001, *OR* = 4.68, indicating proactive facilitation. However, this difference was not significant on the delayed test, *z* ratio = 2.57, *p* =.05, *OR* = 2.41. The other conditional cells showed proactive interference on the immediate test: Changed + List 1 Not Recalled, *z* ratio = 4.72, *p* <.001, *OR* = 0.18, and Not Changed + List 1 Not Recalled, *z* ratio = 3.91, *p* <.001, *OR* = 0.29. On the delayed test, interference remained for the Changed + List 1 Not Recalled cell, *z* ratio = 2.89, *p* =.02, *OR* = 0.37, but was eliminated for the Not Changed + List 1 Not Recalled cell, *z* ratio = 2.15, *p* =.14, *OR* = 0.52.

Collectively, these conditional analyses suggest that the lack of overall proactive memory effects in unconditional List 2 recall reflects offsetting contributions from the conditional cells across delays. Although recollection-based memory for change declined over time, the delayed test also showed reduced interference in the cells lacking change recollection, which offset the fewer instances of facilitation. On the immediate test, recall was highest for changes accompanied by List 1 recall, and the advantage of this cell was further supported by its greater frequency relative to the delayed test. By contrast, the facilitation associated with Changed + List 1 Recalled was not significant on the delayed test, consistent with the assumption that proactive facilitation becomes weaker as recollection-based memory for changes diminishes. Unlike the pattern of retroactive effects, on the delayed test, the intermediate change memory cell was associated with interference, whereas recall for the cell without memory for change did not differ from the control cell. However, these two cells did not significantly differ from each other, suggesting that their underlying processes may partially overlap. This similarity likely reflects the fact that participants can exhibit change awareness at test without having accurately retrieved List 1 responses during List 2 study, meaning the intermediate cell likely contains a mixture of items with and without genuine study-phase retrievals. Consequently, competitor accessibility in the Changed + List 1 Not Recalled cell may not be uniformly increased relative to the Not Changed cell, attenuating the potential to observe differences between the two. Collectively, the pattern of results suggests that the absence of recollection was the primary factor associated with whether proactive interference occurred on the delayed test.

#### Intrusions from non-target lists

**Fig. 7:**
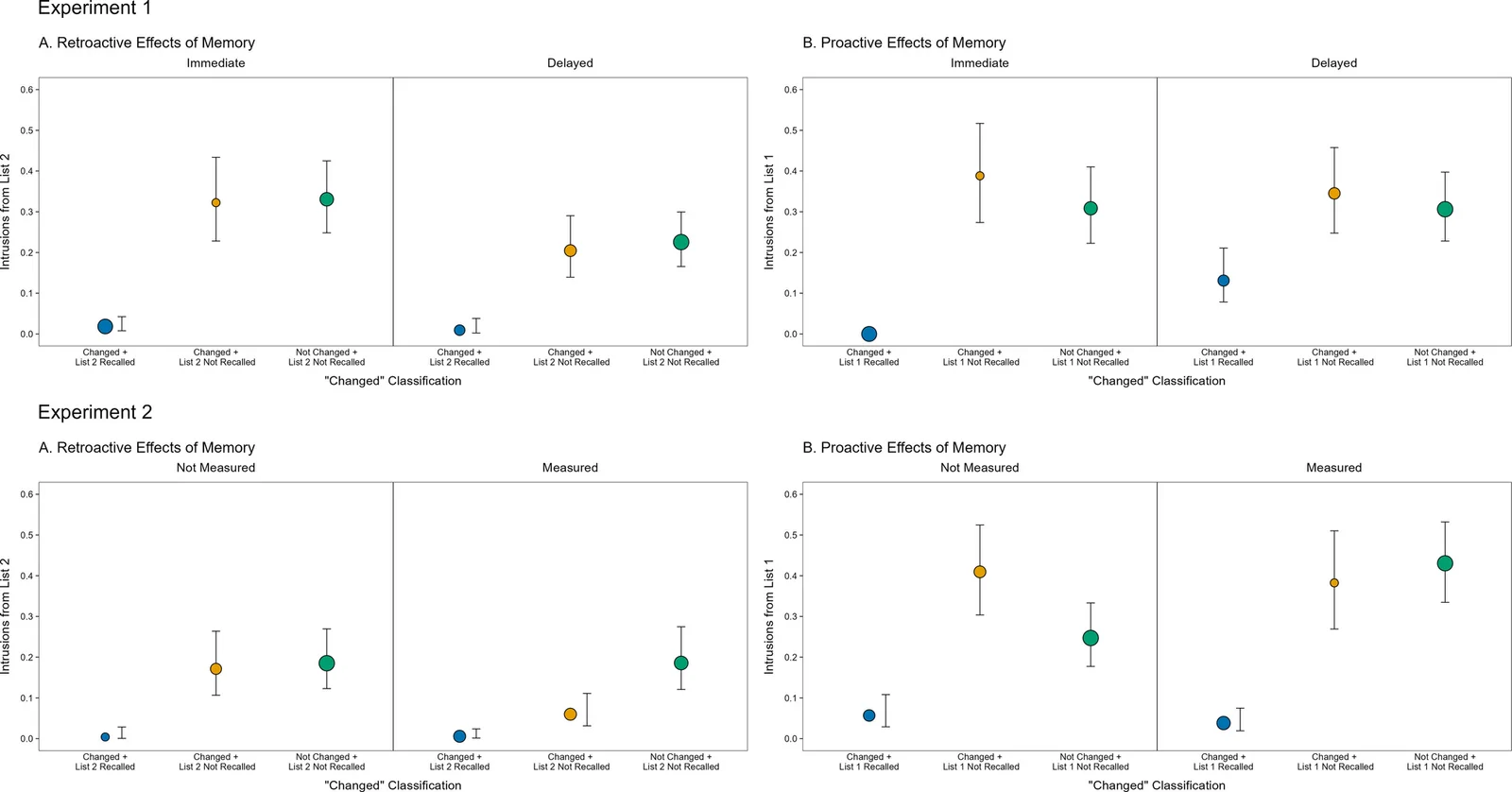
Intrusions for changed items conditionalized on “changed” classifications. Points are probabilities estimated from mixed-effects models. Error bars are 95% confidence intervals. For the immediate test group in Experiment 1b, the estimated probability of the blue point is approximately zero; the corresponding interval spans the full bounded range and is therefore omitted. Point areas indicate relative differences in the proportions of observations in each cell. Those proportions are displayed in Table 2

We expected that intrusion rates would be determined partly by change recollection probabilities, as change recollection entails recalling non-target list response and thus negates intrusions on many occasions. As for correct recall, we assumed that these instances occurred primarily for items that elicited change detection and study-phase retrievals of List 1 responses during List 2 study, promoting integrative encoding. In both retroactive and proactive cases, resultant integrated representations support later retrieval of contextual details (e.g., the temporal order of responses), aiding correct recall and reducing intrusion rates (Jacoby et al., 2015). When change awareness occurs without recollection or is absent altogether, our expectations for intrusion rates differ across retroactive and proactive situations and are therefore addressed in their respective sections. Figure 7 (top panels) displays conditional intrusion rates for Experiments 1a and 1b. We conducted separate mixed-effects models for List 2 intrusions (Experiment 1a) and List 1 intrusions (Experiment 1b), each including fixed effects of Classification (“Changed”) with three levels and Test Delay.

**Retroactive effects**

No prior study of retroactive effects has examined intrusion rates conditionalized on “changed” classifications. However, it is reasonable to expect very low intrusions when participants also recall non-target responses (change recollection) and higher intrusions when participants remember changes without recalling List 2 responses or when not remembering changes at all. When changes are remembered without List 2 recall, intrusions are still likely because that cell often reflects instances when the earlier experience of detecting change did not produce successful integration but did encode lower fidelity attributes of change.

The model of conditional List 2 intrusions yielded a significant effect of Test Delay, χ^2^ (1) = 7.78, *p* <.01, *OR* = 0.54, showing lower intrusions on the delayed than on the immediate test, and a significant effect of Classification, χ^2^ (2) = 74.71, *p* <.001, showing lower intrusions when changes were recollected than for the other two conditional cells, smallest *z* ratio = 7.38, *p* <.001, *OR* = 0.04, which did not significantly differ, *z* ratio = 0.44, *p* =.90, *OR* = 0.92. The interaction was not significant, χ^2^ (2) = 0.08, *p* =.96. Intrusions were near floor when changes were recollected, especially on the immediate test, suggesting that effective change memory counteracted interference. In contrast, intrusions in the other cells declined after a delay, consistent with reduced retroactive interference as competing representations became less accessible over time.

**Proactive effects**

In contrast to retroactive effects, prior work shows more intrusions when participants remember changes without recollecting them compared to when they do not remember changes at all (Garlitch & Wahlheim, 2020b; Wahlheim et al., 2025). Awareness of changes without recalling competitors may still be a downstream consequence of earlier retrieval and comparison between original and changed responses, thus leading the original responses to be more accessible at test. Thus, when changes are not recollected, List 1 intrusions may be higher when participants are aware versus unaware of changes at test.

The model of conditional List 1 intrusions yielded a significant effect of Classification, χ^2^ (2) = 19.54, *p* <.001, but no significant effect of Test Delay, χ^2^ (1) = 0.14, *p* =.70, *OR* = 0.003, and no significant interaction, χ^2^ (2) = 0.38, *p* =.83. Despite the effect of Classification, pairwise comparisons collapsing over delay were not significant, largest *z* ratio = 1.45, *p* =.31, *OR* = 1.30, which likely reflects high uncertainty around the near-zero estimate in the Changed + List 1 Recalled cell on the immediate test. Nevertheless, as with retroactive effects, intrusions were nominally lowest in that Classification cell representing change recollection, which occurred more frequently on the immediate than on the delayed test. However, unlike earlier findings, intrusions were not higher when changes were remembered but not recollected (Changed + List 1 Not Recalled) than when changes were not remembered at all (Not Changed + List 1 Not Recalled). Collectively, these results indicate that the overall higher intrusions for delayed than immediate tests reported earlier reflect fewer instances of change recollection to mitigate interference and comparable magnitudes of interference over time when changes were not recollected.

#### Summary of Experiments 1a and 1b

Collectively, these results replicate shifts in retroactive and proactive memory effects over time while clarifying the role of memory for change. In the unconditional analyses, retroactive interference was evident on the immediate test in reduced List 1 correct recall for changed items, but this difference was eliminated at delay, and retroactive intrusions did not increase over time. In contrast, proactive interference was not apparent in overall List 2 correct recall, but was evident in intrusion rates, which were higher on the delayed than on the immediate test. Conditional analyses showed that these overall patterns reflected shifting mixtures of facilitation and interference across change-memory states. Change recollection, more frequent on the immediate test, was associated with retroactive and proactive facilitation in correct recall; however, proactive facilitation was reduced at delay as recollection declined. By contrast, retroactive facilitation was larger at delay, offsetting interference and eliminating the overall effect. Conditional intrusion analyses converged with these findings in showing that concurrent change awareness and recollection most reliably reduced intrusions, whereas the absence of recollection left interference relatively unmitigated over time.

### Experiments 2a and 2b

Experiments 1a and 1b characterized the role of memory for changes in retroactive and proactive memory effects over time. However, the role of detecting changes during study could only be inferred indirectly from test measures of memory for change. Experiments 2a and 2b addressed this limitation by directly manipulating whether participants were required to overtly detect changes and recall List 1 responses during List 2 study. Importantly, these experiments included only delayed tests to increase sensitivity to potential effects of overt change detection and study-phase retrieval, as retrieval practice effects are often larger after a delay (Rowland, 2014). Predictions about the consequences of requiring change detection and study-phase retrieval can be derived from prior work manipulating controlled detection of changes. Instructions to look for changes produced retroactive and proactive facilitation (Jacoby et al., 2015), whereas other work found that including a detection measure did not moderate proactive memory effects, likely because participants spontaneously engaged in change detection and study-phase retrieval even when uninstructed (Wahlheim & Jacoby, 2013).

Retroactive and proactive memory effects on delayed tests may also be differentially affected by overt detection and study-phase retrieval. When those measures are used, retroactive paradigms entail retrieval practice of the target-list response, whereas proactive paradigms entail retrieval of the non-target response. Practicing retrieval of the target response should confer clear downstream benefits at final test. In contrast, retrieving the non-target response can support integrative encoding of List 1 and List 2 responses, thereby enhancing memory for the List 2 response, but it may also increase intrusions of List 1 responses at test due to their heightened accessibility. Thus, the downstream benefits of overt study-phase measures may be more apparent under retroactive than proactive conditions, with the latter including more balanced tradeoffs between memory improvement and impairment. Finally, including these measures allowed us to conditionalize final cued recall to jointly characterize the roles of change awareness during study (detection) and at test (memory for change) for the first time using a delayed testing procedure. The theoretical implications of these double-conditional analyses are considered in greater detail when presenting hypotheses immediately before those results.

#### Overall cued-recall performance

##### Correct recall from target lists

We assessed retroactive and proactive memory effects by comparing correct recall from target lists for changed and control items in Experiments 2a and 2b (Fig. 3, bottom panels), respectively. We tested for differences between item types across change-detection groups using separate models for List 1 recall (retroactive effects) and List 2 recall (proactive effects), with fixed effects of Item Type (Control vs. Changed) and Change Detection (Measured vs. Not Measured). As described above, the change-detection measure also entailed overt attempts to retrieve List 1 responses when participants indicated that pairs included cues that repeated and responses that changed.

**Retroactive effects**

The model of List 1 recall yielded no significant effect of Change Detection, χ^2^ (1) = 2.16, *p* =.14, *OR* = 0.70, but there was a significant effect of Item Type, χ^2^ (1) = 40.49, *p* <.001, *OR* = 0.38, and a significant interaction, χ^2^ (1) = 14.10, *p* <.001. List 1 recall was not significantly different between item types when change detection was not measured, *z* ratio = 1.93, *p* =.05, *OR* = 0.77, replicating delayed-test results from Experiment 1a. However, List 1 recall was significantly higher for changed than control items when change detection was measured, *z* ratio = 7.15, *p* <.001, *OR* = 2.65, showing a substantial retroactive facilitation effect in overall cued recall.

**Proactive effects**

The model for List 2 recall yielded a significant effect of Change Detection, χ^2^ (1) = 5.37, *p* =.02, *OR* = 0.54, showing lower recall when change detection was measured, but there was no significant effect of Item Type, χ^2^ (1) = 2.21, *p* =.14, *OR* = 1.58, and no significant interaction, χ^2^ (1) = 0.76, *p* =.38. This effect may reflect the division of attention between List 1 retrieval and List 2 encoding incurred by the change-detection task, which would impair encoding of the List 2 responses. However, because this was a between-subjects design, this may also reflect inherent group differences for which we could not account. Importantly, the absence of proactive facilitation or interference suggested that retrieval practice of List 1 responses created a trade-off of improvement and impairment that may only be revealed using conditional analyses.

##### Intrusions from non-target lists

We next examined retroactive and proactive memory effects by comparing intrusions from non-target lists for changed items within Experiments 2a and 2b (Fig. 4, bottom panels), respectively. We tested for differences across change-detection groups using separate models for List 2 intrusions (retroactive effects) and List 1 intrusions (proactive effects), each with a fixed effect of Change Detection.

**Retroactive effects**

The model of List 2 intrusions yielded a significant effect of Change Detection, χ^2^ (1) = 6.93, *p* <.01, *OR* = 0.59. This result shows that the reversal of retroactive interference when change detection was measured improved memory accuracy not only by enhancing correct recall, reported above, but also by decreasing the probability of intrusions.

**Proactive effects**

The model of List 1 intrusions yielded no significant effect of Change Detection, χ^2^ (1) = 0.48, *p* =.49, *OR* = 0.86, indicating that this type of proactive interference did not differ based on whether participants overtly engaged in change detection and List 1 retrieval attempts. This result coincides with the finding that the inclusion of the change-detection measure did not result in proactive facilitation or interference in overall correct recall, and further bolsters the importance of considering conditional analyses to fully characterize these effects.

##### Summary

Together, these results show that including the change-detection measure selectively influenced overall retroactive effects, reversing interference to facilitation and reducing intrusions. Although there was no evidence of such influence on overall proactive effects, conditional analyses are likely to reveal tradeoffs between facilitation and interference.

#### “Changed” classifications at test

Following the approach from Experiments 1a and 1b, we next examined change awareness at the time of test to characterize the observation differences in subsequent conditional analyses. Table 1 (right columns) displays the probabilities of the three “changed” classifications described above. We again modeled the two classification levels implying change awareness at test. Because the change-detection measure increases non-target response accessibility in the proactive case, we expected changes to be better recalled in that condition and no such differences in the retroactive case, which entails divided attention during encoding of changes.

##### Response probabilities for “changed” classifications

For both Experiment 2a and 2b, mixed-effects models with fixed effects of Test Classification (Changed + Non-Target Recalled vs. Changed + Non-Target Not Recalled) and Change Detection and by-subject random intercepts exhibited singular fits. As in the prior experiments, we instead conducted two separate regression models with the same predictors, using subject-clustered robust standard errors for follow-up analyses. Model-implied probabilities for each combination of Test Classification and Change Detection are displayed in Fig. 5 (bottom panels).

**Retroactive effects**

The model for retroactive effects yielded no significant effect of Test Classification, *F*(1, 78) = 2.72, *p* =.10, *η*^*2*^*p* =.034, Change Detection, *F*(1, 78) = 0.86, *p* =.36, *η*^*2*^*p* =.011, or their interaction, *F*(1, 78) = 0.95, *p* =.33, *η*^*2*^*p* =.012. These results replicate the lack of differences on the delayed test in Experiment 1a and indicate that including a change-detection measure did not substantially affect non-target accessibility at test in the retroactive case.

**Proactive effects**

The model for proactive effects yielded no significant effect of Test Classification, *F*(1, 77) = 0.10, *p* =.76, *η*^*2*^*p* =.001, Change Detection, *F*(1, 77) = 3.91, *p* =.05, *η*^*2*^*p* =.048, or their interaction, *F*(1, 77) = 2.77, *p* =.10, *η*^*2*^*p* =.035. Although the effect of Change Detection was not significant, visual inspection of Fig. 5B suggests that it was primarily carried by classifications that involved correct List 1 recall (i.e., change recollection), but the interaction needed to confirm this was not obtained. These results thus provide inconclusive evidence that overt change detection and study phase retrievals selectively improved recollection-based classifications of change; further validation in subsequent work is required to confirm this assertion.

#### Correct recall for changed items conditionalized on “changed” classifications at test

Here, we examine how the overall retroactive and proactive memory effects observed in each change-detection group varied with different levels of change awareness at test, using the same approach as in the prior experiments. For analyses of correct recall from target lists, we included all three levels of “changed” classifications and control items; for analyses of intrusions from non-target lists, we included only the three levels of “changed” classifications. The predictions in Fig. 2B apply here as well, but we expect that overt change detection will lead to retroactive facilitation when changes are remembered, even when the alternative-list responses were not recalled. This outcome is assumed to reflect the retrieval practice benefit for target-list responses that study-phase retrievals promote. It would also verify that retrieval practice alone is sufficient to produce this benefit, without requiring integration of both responses.

##### Correct recall from target lists

Figure 6 (bottom panels) shows conditional correct recall from target lists for Experiments 2a and 2b, respectively. We tested for differences between “changed” classifications and control items across change-detection groups for each test type using separate models for List 1 recall (Fig. 6A) and List 2 recall (Fig. 6B), each including fixed effects of Classification (all three “Changed” classifications + control items) and Change Detection. For each test type, we first report differences among the three levels of “changed” classifications before examining differences between changed and control items, which characterize retroactive and proactive memory effects associated with memory for change.

**Retroactive effects**

Prior investigations of retroactive effects using change-detection measures and immediate tests have shown that change awareness supports retroactive facilitation regardless of whether those changes are later recollected (Jacoby et al., 2015; Negley et al., 2018). In similar situations without change-detection measures, non-recollective change awareness has been associated with intermediate memory performance (Garlitch & Wahlheim, 2020a). Therefore, we expected non-recollective change awareness to produce facilitation only when change detection was measured, thus reflecting primarily the influence of study-phase retrieval practice on increased List 1 response accessibility. Because change recollection was associated with retroactive facilitation in all these studies and Experiment 1a, we also expected to observe facilitation when changes were recollected in both detection groups.

The model of List 1 recall yielded no significant effect of Change Detection, χ^2^ (1) = 1.64, *p* =.20, *OR* = 0.64, but the effect of Classification, χ^2^ (3) = 126.50, *p* <.001, and the interaction, χ^2^ (3) = 19.77, *p* <.001, were both significant. List 1 recall differed significantly across classification types: it was highest for Changed + List 2 Recalled, intermediate for Changed + List 2 Not Recalled, and lowest for Not Changed + List 2 Not Recalled, with all pairwise differences being significant, smallest *z* ratio = 4.96, *p* <.001, *OR* = 2.29. Pairwise comparisons showed significantly higher recall in the Changed + List 2 Recalled than control cell regardless of whether change detection was measured, smallest *z* ratio = 6.46, *p* <.001, *OR* = 9.60, indicating retroactive facilitation in both instances. In contrast, recall was only higher in the Changed + List 2 Not Recalled than control cell when change detection was measured, *z* ratio = 6.37, *p* <.001, *OR* = 3.65; when change detection was not measured, no significant effect was observed, *z* ratio = 0.17, *p* =.99, *OR* = 1.04. Finally, there was no significant difference between Not Changed + List 2 Not Recalled and the control cell in both detection groups, largest *z* ratio = 1.72, *p* =.32, *OR* = 0.75. Taken together, these results suggest that the reversal of retroactive interference to facilitation shown in overall cued recall included contributions from retrieval practice of List 1 responses promoted by overt change detections and integrative encoding opportunities that promoted subsequent test-phase change recollection.

**Proactive effects**

When change detection was not measured in the proactive case, we expected to replicate conditional correct recall results from prior work (e.g., Wahlheim & Jacoby, 2013) and the delayed test in Experiment 1b, both showing facilitation when changes were recollected and interference in the other conditional cells. Additionally, we expected that the influence of the change-detection measure on the frequencies of “changed” classifications would determine whether these effects persisted across detection groups; however, we had no clear expectations for conditional proactive effects when change detection was measured given the expected tradeoffs between facilitation and interference described earlier.

The model of List 2 recall yielded a significant effect of Change Detection, χ^2^ (1) = 9.17, *p* <.01, *OR* = 0.49, showing that List 2 recall was significantly lower when change detection was measured, and Classification, χ^2^ (3) = 114.56, *p* <.001, but there was no significant interaction, χ^2^ (3) = 1.52, *p* =.68. List 2 recall differed significantly across classification types: it was higher for Changed + List 1 Recalled than in the other two conditional cells, smallest *z* ratio = 8.65, *p* <.001, *OR* = 7.58, which were not significantly different, *z* ratio = 0.77, *p* =.87, *OR* = 0.85. Pairwise comparisons indicated that recall was significantly higher in the Changed + List 1 Recalled than the control cell, *z* ratio = 2.70, *p* =.04, *OR* = 2.31, showing a proactive facilitation effect. In contrast, recall was significantly lower in the Changed + List 1 Not Recalled and Not Changed + List 1 Not Recalled cells than the control cell, smallest *z* ratio = 3.39, *p* <.01, *OR* = 0.36, showing proactive interference effects. Although the inclusion of the change-detection measure was associated with nominally higher List 1 (non-target) recollection at test, the observation difference was too subtle to offset diminished List 2 recall associated with the other correction classifications when recollection-based retrieval was absent. These findings characterize the trade-off of effects underlying the overall decrease in List 2 recall when the change-detection measure was included in List 2 study. Notably, the similar degree of interference across the cells without change recollection again likely reflects that test-phase change awareness does not guarantee prior study-phase retrieval, as discussed above.

##### Intrusions from non-target lists

We next examined intrusion rates conditionalized on levels of memory for change across change-detection groups (Fig. 7, lower panels). When change detection was not measured, we expected to replicate the delayed-test results from Experiments 1a (retroactive effects) and 1b (proactive effects), both of which showed intrusion rates were lowest when changes were recollected at test. We also expected this effect to persist when change detection was measured, as test-phase change recollection should nearly eliminate intrusions. For the other two conditional cells, our expectations differ between retroactive and proactive cases and are therefore discussed in their respective sections. We followed the analytic approach from the previous conditional analyses, conducting separate mixed-effects models for List 2 intrusions (Experiment 2a) and List 1 intrusions (Experiment 2b), each including fixed effects of Classification (“Changed”) with three levels and Change Detection.

**Retroactive effects**

In the retroactive case, retrieval practice of List 1 (target) responses promoted by the change-detection measure disrupts List 2 (non-target) encoding. Consequently, test-phase change awareness should be less likely to produce intrusions when detection is measured than when it is not. We therefore expected intrusion rates associated with intermediate change awareness to be lower when detection was measured versus when it was not. By contrast, we did not expect this effect to extend to change recollection because intrusions are at floor when changes are recollected at test, except in very rare cases when participants report the same response twice unusually often, typically reflecting uncertainty and guessing.

The model of conditional List 2 intrusions yielded no significant effect of Change Detection, χ^2^ (1) = 2.73, *p* =.10, *OR* = 1.32, a significant effect of Classification, χ^2^ (2) = 40.21, *p* <.001, and a significant interaction, χ^2^ (2) = 8.12, *p* =.02. When change detection was not measured, the probability of a List 2 intrusion differed significantly across classification types: it was lower in the Changed + List 2 Recalled cell than the other two conditional cells, smallest *z* ratio = 3.90, *p* <.001, *OR* = 0.02, which did not differ, *z* ratio = 0.36, *p* =.93, *OR* = 0.91. In contrast, when detection was measured, the probability of a List 2 intrusion differed across classification types in the following way: Changed + List 2 Recalled < Changed + List 2 Not Recalled < Not Changed + List 2 Not Recalled, with all pairwise differences being significant, smallest *z* ratio = 3.18, *p* <.01, *OR* = 0.09. The interaction showed that intrusion rates in the Changed + List 2 Not Recalled cell were significantly lower when change detection was measured than when it was not, *z* ratio = 3.25, *p* <.01, *OR* = 0.31. Intrusion rates associated with the other cells were not significantly different across change-detection groups, largest *z* ratio = 0.29, *p* =.77, *OR* = 0.71. These results suggest that the overall reduction in intrusions associated with the inclusion of the change-detection measure was a consequence of encoding operations that improved memory for List 1 and List 2 responses and enabled rejection of intrusions.

**Proactive effects**

In Experiment 1b, we expected higher intrusion rates for intermediate memory for change compared to a lack of memory for change; however, this effect was not observed significantly, despite the obvious nominal trend. Following the same reasoning outlined earlier, we expected to observe this effect here when change detection was not measured. However, we had no clear expectations for when change detection was measured, as the outcome will depend on how the measure affects the relative frequencies of “changed” classifications.

The model of conditional List 1 intrusions yielded no significant effect of Change Detection, χ^2^ (1) = 2.59, *p* =.11, *OR* = 0.90, but there was a significant effect of Classification, χ^2^ (2) = 90.78, *p* <.001, and a significant interaction, χ^2^ (2) = 10.08, *p* <.01. When change detection was not measured, the probability of a List 1 intrusion differed across classification types in the following way: Changed + List 1 Recalled < Not Changed + List 1 Not Recalled < Changed + List 1 Not Recalled, with all pairwise differences being significant, smallest *z* ratio = 3.00, *p* <.001, *OR* = 0.47. The pattern was different when detection was measured: the probability of a List 1 intrusion was significantly lower in the Changed + List 1 Recalled cell than the other two conditional cells, smallest *z* ratio = 6.80, *p* <.001, *OR* = 0.06, which did not differ, *z* ratio = 0.74, *p* =.74, *OR* = 0.82. The interaction showed that, for the Not Changed + List 1 Not Recalled cell, intrusion rates were significantly higher when change detection was measured, *z* ratio = 3.04, *p* <.01, *OR* = 2.30. Intrusion rates for the other two classification conditions did not differ on this basis, largest *z* ratio = 0.86, *p* =.39, *OR* = 1.52. Overall, these results suggest that the lack of differences in overall List 1 intrusion rates across change-detection groups comprises two opposing effects of retrieval practice: the enhanced accessibility of List 1 (non-target) responses reduced intrusion rates when changes were recollected at test, but increased intrusion rates when recollection failed.

### Conditional target-list cued recall for changed items after change detection

In the preceding conditional analyses, we inferred the consequences of change detection and retrieval practice during study on cued recall based on test measures of memory for changes. In the final series of analyses, we took a more focused approach to characterizing those memory consequences by examining correct recall and intrusions from the group who made overt change detections in List 2. This allowed us to restrict analyses of changed items conditionalized on test classifications to the items for which participants recalled List 1 responses during List 2. These are verified instances that provided opportunities for retrieval practice effects and cross-list integrative encoding. Figure 2C summarizes a priori predictions for these analyses. Because study-phase retrieval of the target was verified for all items, we expected to observe retroactive facilitation across all three change-awareness states, reflecting the sufficiency of retrieval practice for increasing the accessibility of target-list responses regardless of whether integration with alternate-list responses occurred. However, such facilitation should be greatest when change is recollected, reflecting the added contribution of integrative encoding. In contrast, because study-phase retrieval increased the accessibility of competing-list responses under proactive conditions, we expected to observe facilitation only when change is recollected, which the account assumes reflects access to temporal contextual features. When memory for changes is not based on recollection or is absent, we expected to observe interference because the heightened accessibility of the competitor from retrieval practice remains the primary basis for reporting responses when temporal contextual features are inaccessible. As in prior conditional analyses, we included control items in models of correct recall, allowing for inferences about interference and facilitation effects, but did not include control items in the intrusion analyses.

#### Correct recall from target lists

**Fig. 8:**
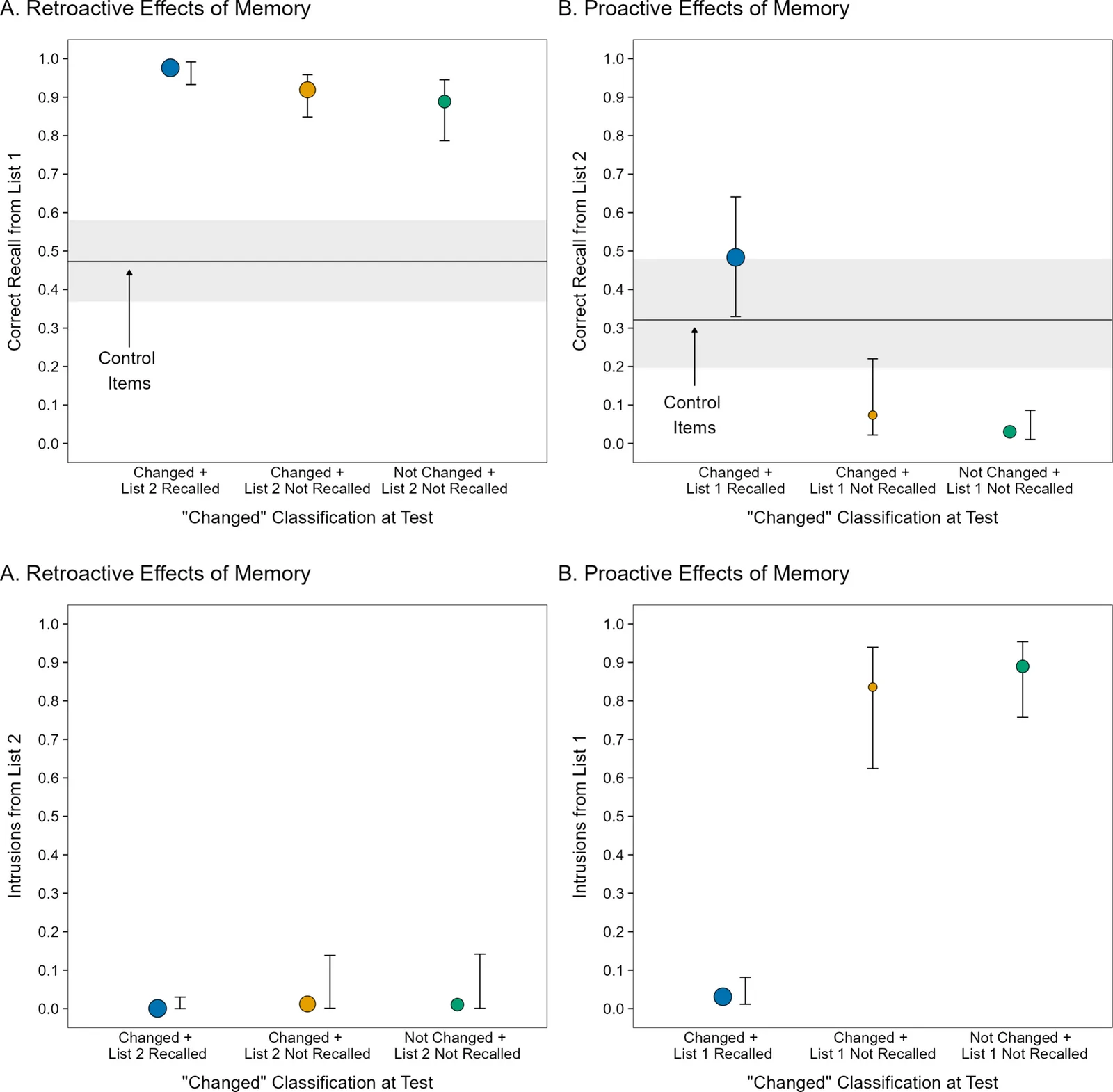
Correct recall and intrusions for changed items detected in List 2 and accompanied by recall of List 1 responses conditionalized on subsequent memory for change in Experiment 2. Points are probabilities estimated from mixed-effects models. Error bars and shaded regions are 95% confidence intervals. The points include observations for which changes were detected and List 1 responses were recalled during List 2 study. Their sizes indicate relative differences in the number of observations per cell within Experiment 2a (top and bottom A panels) and Experiment 2b (top and bottom B panels). Those proportions are displayed in Table 2. The horizontal bars above are model estimates for control items

Figure 8 (upper panels) shows conditional correct recall rates; Table 2 (left column) shows the corresponding proportions of observations in conditional cells. We modeled List 1 recall (retroactive effects) and List 2 recall (proactive effects) separately using models with a fixed effect of Classification with four levels (all three “Changed classifications + control items).

**Table 2:** “Changed” classification proportions on List 2 and at test for changed items in the detection measure groups in Experiment 2

		List 2 Classification		
Target List	Test Classification	Changed +	Changed +	Not Changed +
(Memory Effects)		List 1 Recalled	List 1 Not Recalled	List 1 Not Recalled
Recall List 1	Changed + List 2 Recalled	.29	.01	.01
(Retroactive)	Changed + List 2 Not Recalled	.23	.02	.06
	Not Changed + List 2 Not Recalled	.14	.04	.20
Recall List 2	Changed + List 1 Recalled	.33	.00	.01
(Proactive)	Changed + List 1 Not Recalled	.07	.04	.09
	Not Changed + List 1 Not Recalled	.16	.04	.26

The proportions in the first column correspond to the cell sizes of conditional correct recall probabilities in Fig. 8. The proportions in the middle and right columns are included for completeness

**Retroactive effects**

The analyses of retroactive effects assessed whether retrieval practice of the target response and integrative encoding with the non-target response both contributed to the enhanced List 1 recall on the final test associated with recalling List 1 responses during List 2. If enhanced final List 1 recall is due to retrieval practice alone, then performance should not differ across classifications, because in all three cells, participants had retrieved the List 1 response during List 2. In contrast, if retrieval practice and integrative encoding both contributed, then List 1 recall should be highest when List 2 responses were also recalled at test, which indicated instances when change attributes were most effectively encoded.

The model of List 1 recall yielded a significant effect of Classification, χ^2^ (3) = 133.67, *p* <.001. For changed items, List 1 recall was higher in the Changed + List 2 Recalled cell than in the Not Changed + List 2 Not Recalled cell, *z* ratio = 2.65, *p* =.04, *OR* = 5.17; no other pairwise differences between classification types were significant, largest *z* ratio = 2.16, *p* =.13, *OR* = 3.63. Further pairwise comparisons revealed that List 1 recall was significantly higher across all classification types relative to control items, smallest *z* ratio = 6.18, *p* <.001, showing facilitation effects. Notably, the magnitude of facilitation was larger when changes were recollected at test (blue point, *OR* = 45.99) than when participants exhibited non-recollective change awareness (orange point, *OR* = 12.68) or when participants were not aware of change at test (green point, *OR* = 8.90); this outsized benefit associated with change recollection suggests a contribution of integrative encoding to overall facilitation that was mostly driven by retrieval practice effects.

**Proactive effects**

In contrast to the retroactive case, analyses of proactive effects assessed the extent to which retrieval practice of List 1 (non-target) responses during List 2 improved or impaired final List 2 recall. Because change recollection is often associated with proactive memory benefits, we expected proactive facilitation in recall of List 2 responses when List 1 responses were also recalled at test and interference absent such change recollection.

The model of List 2 recall yielded a significant effect of Classification, χ^2^ (3) = 50.42, *p* <.001. For changed items, List 2 recall was significantly higher for Changed + List 1 Recalled than the other two conditional cells, smallest *z* ratio = 3.94, *p* <.001, *OR* = 11.81, which did not significantly differ from each other, *z* ratio = 1.22, *p* =.61, *OR* = 2.54. Further pairwise comparisons showed that, relative to control items, List 2 recall was not significantly different when changes were recollected at test (blue point), *z* ratio = 1.66, *p* =.35, *OR* = 1.98. In contrast, List 2 recall was significantly lower when changes were not recollected at test (both orange and green points) compared to control items, smallest *z* ratio = 2.57, *p* <.05, *OR* = 0.17, showing proactive interference effects. Although facilitation was not observed, these results suggest that retrieval practice eliminated interference when integrated representations remained intact across the test delay. The absence of facilitation likely reflected diminished quality of integration, at least for some trials, across the delay period. In contrast, when integrated representations were not retained until test or not effectively formed during List 2, retrieval practice of competitors led to proactive interference.

#### Intrusions from non-target lists

Figure 8 (lower panels) shows conditional intrusion rates across retroactive and proactive memory groups. We modeled List 2 intrusion rates (retroactive effects) and List 1 intrusion rates (proactive effects) using separate mixed-effects models, each with a fixed effect of Classification (“Changed”) with three levels.

**Retroactive effects**

The analyses of retroactive effects of memory assessed the extent to which retrieval practice of target responses during the encoding of non-target responses in List 2 counteracted interference by preventing intrusions. If retrieval practice is sufficient to counteract interference in the form of intrusions, cells will not differ based on test-phase classification; if integrative encoding is necessary, cells will differ based on test-phase classification. The model of List 2 intrusions yielded no significant effect of Classification, χ^2^ (2) = 5.86, *p* =.05; intrusion rates were near floor in each classification cell. This result suggests that successful retrieval practice was sufficient for counteracting retroactive interference in the form of intrusions.

**Proactive effects**

The analyses of proactive effects of memory assessed the extent to which retrieval practice of non-target responses while encoding target responses either counteracted or contributed to interference effects in the form of List 1 intrusions. Following the rationale for correct recall above, retrieval practice of List 1 responses in List 2 can increase their accessibility, leading them to be more easily rejected when they and their list context can be recollected or more likely to be reported when highly accessible responses are not accompanied by list context information that could be used as a basis for withholding those responses. We expected intrusion rates to be at floor when List 1 responses were recalled during list 2 and at test, because those instances would entail reporting the List 1 response twice consecutively. Conversely, we expected intrusions to be very high in the other cells because those instances would be mostly devoid of recollective content that could oppose the accessibility of List 1 responses heightened by verified successful retrieval practice.

The model of List 1 intrusions yielded a significant effect of Classification, χ^2^ (2) = 64.77, *p* <.001. The probability of an intrusion from List 1 differed across classification types in the following way: it was significantly lower when change was recollected (blue point) than the other two cells when change was not recollected (orange and green points), smallest *z* ratio = 6.61, *p* <.001, *OR* = 0.01, which did not significantly differ from each other, *z* ratio = 0.70, *p* =.76, *OR* = 0.63. Consistent with conditional correct recall, these results suggest that the increased accessibility of List 1 responses via retrieval practice led to more interference when list-context information was not recollected, with almost no interference when the experience of change was preserved in memory.

#### Summary of Experiments 2a and 2b

The results show that change detection and associated retrieval practice differentially affected memory performance on delayed tests across interference types and states of change awareness. In the unconditional analyses, retroactive facilitation occurred only when change detection was measured, and intrusion rates were lower in this condition than when detection was not measured. By contrast, no proactive effects emerged in correct recall, and intrusion rates did not differ based on whether detection was measured. However, including the detection measure increased recollection-based memory for changes only in the proactive case, which entails correct recall of List 1 responses. Collectively, these results show that the retrieval practice stimulated by the detection measure promoted target list recall more directly under retroactive than under proactive memory conditions, suggesting that the latter reflected a mixture of improvement and impairment resulting from the heightened accessibility of List 1 responses.

Analyses conditionalized on memory for changes at test further characterized these effects. For retroactive effects, change recollection was associated with facilitation and reduced intrusion rates, regardless of whether detection was measured. Memory for changes not based on recollection were also associated with retroactive facilitation only when detection was measured; intrusion rates for this cell were also lower when detection was measured than when it was not. Together, these results show that retrieval practice benefits were spread across the two types of memory for change, suggesting that the retrieval overtly stimulated during study led additional items to acquire change attributes that were later associated with better target-list memory, even in the absence of recalling the changed responses. For proactive effects, regardless of whether detection was measured, facilitation occurred when changes were recollected and interference occurred when recollection failed, thus revealing the tradeoff that led to an absence of proactive effects in overall correct recall. Unsurprisingly, intrusion rates were lower when changes were recollected than in the other two conditionalized cells. These results suggest that retrieval practice of non-target responses improved memory accuracy when change attributes were preserved in memory until test and impaired memory accuracy otherwise, presumably because competitors remained accessible, but their list context could not be recollected.

Finally, the consequences of retrieval practice when detection was measured were revealed more directly by analyses of correct recall and intrusions only for changed items with verified List 1 retrievals during List 2 conditionalized on the types of memory for change at test. For retroactive effects, the finding that facilitation, which largely reflected retrieval practice benefits, was greatest when changes were recollected suggested that integrative encoding also contributed to such facilitation, albeit to a minor degree. By contrast, retrieval practice alone was sufficient to minimize intrusion-based interference, which was minimal and did not differ across types of memory for change. For proactive effects, change recollection was critical for avoiding interference following successful retrieval practice, but successful retrieval practice led to sizable interference in correct recall and extremely high intrusion rates when such recollection failed. Collectively, these results starkly illustrate how retrieval practice of targets and competitors that enables the encoding of change attributes produces substantially different mixtures of improvement and impairment across retroactive and proactive memory conditions.

## General discussion

The present study examined the roles of change detection during study and recollection of prior changes at test in retroactive and proactive memory effects. Retroactive effects of List 2 changes on memory for List 1 responses produced interference on the immediate test that was eliminated or reversed after a delay. In contrast, proactive effects of List 1 associations on memory for List 2 responses produced more interference after a delay than on the immediate test. Conditional analyses showed that these overall patterns depended on whether changes were later recollected at test. When change recollection occurred, interference from changed responses was reduced, consistent with the view that change detection and retrieval during List 2 supported encoding of associations between responses. Overt change detection amplified these effects. Retrieving List 1 responses during List 2 improved later memory for List 1 in retroactive conditions but increased the accessibility of competing List 1 responses under proactive conditions. Together, these findings suggest that retrieval practice during List 2 increased the accessibility of List 1 responses while also promoting integrative encoding between responses. These processes had different consequences across retroactive and proactive conditions. Increased accessibility benefited performance when the retrieved response was later the target (retroactive), but it increased interference when it was the competitor (proactive). Both outcomes depended on whether change information remained accessible at test. Table 3 summarizes these patterns of correct recall across experiments, conditions, and levels of analysis.

**Table 3:** Summary of observed retroactive and proactive memory effects in target-list recall across conditions and levels of analysis

	Retroactive			Proactive		
Level of Analysis	*Immediate*	*Delayed*	*Delayed*	*Immediate*	*Delayed*	*Delayed*
	*No Detection*	*No Detection*	*Detection*	*No Detection*	*No Detection*	*Detection*
*Unconditional*	RI	null	RF	null	null	null
*Conditionalized on change awareness (test)*						
Recollected	RF	RF	RF	PF	null	PF
Remembered, not recollected	RI	null	RF	PI	PI	PI
Not remembered	RI	RI	null	PI	null	PI
*Verified study-phase retrievals (in List 2) conditionalized on change awareness (test)*						
Recollected	—	—	RF	—	—	null
Remembered, not recollected	—	—	RF	—	—	PI
Not remembered	—	—	RF	—	—	PI

All comparisons are relative to control (A-B, C-D) items. RF = retroactive facilitation; RI = retroactive interference; PF = proactive facilitation; PI = proactive interference; null = no significant difference from control items. Em dashes (—) indicate conditions not applicable to that analysis level

These findings can be understood in relation to theories of associative interference, which explain how associations formed at different times affect access to one another. Early accounts explained interference in terms of unlearning and response competition (Melton & Irwin, 1940), while later views emphasized interference as reflecting differences in relative trace strengths (Baddeley, 1976) and contextual cues that differentiate memories across time (Mensink & Raaijmakers, 1988). These views are supported by studies showing that contextual changes that isolate memories reduce interference (for a review, see Smith & Vela, 2001). However, many such studies did not consider the role of study-phase retrieval in interference reduction. One notable exception using the list-method-directed forgetting paradigm showed that directional associations from List 2 to List 1 reduced retroactive interference by promoting study-phase retrieval of List 1 items (Sahakyan & Goodmon, 2007). Such findings are consistent with work showing that associative relationships promote retrieval of earlier events, as reflected in better memory for relative order for related than unrelated words (e.g., Winograd & Soloway, 1985) and in studies showing that semantic associations and instructions to notice relationships counteract interference (e.g., Barnes & Underwood, 1959; Postman & Gray, 1977). More recent work has shown that change detection and retrieval practice can counteract interference on immediate tests by enhancing memory for retrieved associations and providing opportunities to integrate changed events (Wahlheim et al., 2021), with retroactive facilitation increasing over time primarily for events that maintained attributes of change (Wahlheim et al., 2026). The present results extend that work by showing that changes in both retroactive and proactive memory effects across delays can be understood by jointly considering the effects of retrieval practice and decreases in memory for changes. Critically, these effects depended on whether integrated representations remained accessible at test.

Beyond clarifying the mechanisms underlying these effects, the present study directly compared retroactive and proactive memory effects within the same experiments while measuring memory for changes and response accessibility from both lists. This builds upon prior work that characterized the roles of retrieval practice and change recollection in retroactive and proactive effects across separate experiments while measuring memory for changes and response accessibility only for target lists (Jacoby et al., 2015). That study showed that controlled retrieval practice could lead to either memory updating or interference depending on subsequent change recollection. However, whether memory updating reflected integrative encoding could only be inferred from subjective reports of remembering changes.

In contrast, the current procedures enabled analyses of response dependencies that more directly assessed the extent to which B and D responses became associated during List 2. Regardless of which list served as the target, recall of target-list responses was enhanced when changes were remembered and responses from the other list were also recalled. When study-phase retrieval and change detection were verified, this benefit was observed for List 1 recall beyond the effects of practicing List 1 retrieval alone. Specifically, memory enhancement for List 1 responses associated with verified A-B retrieval during List 2 was greater when List 2 responses were also recalled at test. Retrieval practice of List 1 responses also increased memory errors for List 2 recall, which were offset only when participants recollected both the List 1 responses and that those responses had changed in List 2. Collectively, the present findings verify the inference drawn across studies that retrieving List 1 responses during List 2 study sometimes produced integrative encoding that bi-directionally benefited memory updating. This bi-directionality likely reflected the engagement of elaborative, semantic processing while retrieved responses were compared to studied items. Moreover, requiring such retrievals may have promoted elaborative encoding (cf. Carpenter, 2009), in addition to simply increasing the frequency of their occurrence (cf. Jacoby et al., 2015). Future studies may explore the extent to which intentional study-phase retrievals and the type of encoding processing engaged drive the balance of these quantitative and qualitative benefits for associative memory representations.

The present findings also have implications for theories of interference. Although trace-strength accounts explain aggregate patterns of temporally dynamic interference effects, they do not include mechanisms necessary to fully account for the present findings. These theories assume that interference between representations of related episodes should decrease to the extent that memory attributes associated with one event outcompete those associated with the other at test. Under this view, co-activation of responses during encoding of the second event should weaken the decisiveness with which one memory outcompetes the other, contributing to interference on both immediate and delayed tests. In contrast, change recollection, reflecting prior co-activation, was associated with retroactive and proactive facilitation on immediate tests, retroactive facilitation on delayed tests, and avoidance of substantial proactive interference on delayed tests. These results suggest that a more complete account of temporal changes in interference should recognize that co-activation can promote dependency between memories for related episodes, thereby moderating proactive and retroactive effects over time. In the retroactive case, retrieval practice and response integration each contributed to reductions in interference over time, although retrieval practice likely played a larger role because the benefits of target-response retrieval increase with delay, whereas degraded change attributes are less consequential for target accessibility and intrusion likelihood. In the proactive case, as interference increased over time, prior retrieval practice of non-target responses increased their accessibility while memory for change attributes diminished, leaving the strength of those competitors as a basis for selecting and reporting those responses (cf. Bennion et al., 2024). Consequently, the extent that memory for change attributes degraded determined the degree of interference, highlighting the importance of integrative encoding processes that support recollection of change.

Beyond the interference literature, a broader body of work using similar stimulus configurations has also assumed that elements from separate episodes can become associated in memory and has suggested that integrative processes support interdependent representations and subsequent memory. This perspective is closely aligned with the present framework, which proposes that retrieval practice during encoding can promote integration across episodes. Prior studies have shown substantial memory benefits for associations within complex arrangements that can be integrated beyond cue-target pairings. For example, several studies have shown that participants remember “closed-loop” configurations such as A-B, B-C, and A-C better than “open-loop” configurations such as A-B, B-C, and C-D (Horner & Burgess, 2014; Horner et al., 2015; Ngo et al., 2019). Moreover, associations within closed-loop structures are more interdependent than those within open-loop structures. Relatedly, semantic relationships between targets (such as B and D) associated with the same cue can reduce interference or even produce facilitation while increasing interdependence (e.g., Antony et al., 2022; Bennion et al., 2024), suggesting that pre-existing relationships between B and D may effectively “close the loop” and integrate otherwise separate associations. Although these studies do not explicitly examine the role of study-phase retrieval in producing such interdependence, the stimulus structures strongly imply that retrieval during encoding could contribute to associating elements across episodes. In this respect, these findings converge with the present results in showing that integrative encoding can support memory updating for complex stimulus configurations.

The present account also aligns with neurocomputational perspectives on hippocampal function by specifying how study-phase retrieval may correspond to interactions between pattern completion and pattern separation. When a cue (A) elicits retrieval of a prior response (B) during A-D study, this is consistent with pattern completion, which reinstates stored representations from partial input (McClelland et al., 1995; Norman & O’Reilly, 2003). This reinstatement can increase the accessibility of prior responses, thereby increasing the likelihood of interference when those responses later compete at test. At the same time, encoding of the A-D association may involve pattern separation, which manifests as the overt detection of changed features, supporting the formation of distinct representations that preserve information about the temporal relationship between responses (Yassa & Stark, 2011). From this perspective, the opposing effects of study-phase retrieval that depend on change awareness may also reflect the extent to which A-B and A-D associations were encoded distinctively while at the same time becoming associated with a common mental context that includes information about the fact that the responses changed. Thus, the consequences of these hippocampal operations during study may reflect whether integrated representations including such changes remain accessible at test.

This interpretation is broadly consistent with evidence that neural reactivation of prior memories during new learning predicts reduced interference and promotes integration across overlapping events (e.g., Chanales et al., 2019; Kuhl et al., 2010). Extending this work, the present findings suggest that the consequences of such reactivation depend on whether retrieval-driven integration supports later access to change information that can guide response selection. This information could be encoded as a type of context akin to metadata about the subjective experience of prior retrieval. A key prediction is that neural measures of reactivation during A-D study should relate not only to subsequent memory but also to the conditional pattern observed here, predicting facilitation when change is later recollected and interference when it is not. Testing this prediction will require combining controlled interference paradigms with methods capable of resolving hippocampal subfields that putatively support pattern completion and separation (Kirwan & Stark, 2007). Taken together, the current patterns of conditional results provide specific constraints on how retrieval-driven changes in accessibility and integration must be implemented in cognitive and neural models.

To conclude, by examining retroactive and proactive effects across delays within the same design, the present results show that study-phase retrieval increases the accessibility of prior responses while also supporting the formation of integrated representations. Critically, whether these processes facilitate or impair later performance depends on the role of the retrieved response at test and on the persistent accessibility of change attributes. These findings refine theoretical accounts of associative interference by showing that retrieval-related increases in accessibility and integrative encoding determine whether prior learning supports or disrupts subsequent remembering. More broadly, they suggest that memory updating reflects a dynamic interaction between retrieval and encoding processes whose consequences unfold across retention intervals that increase the benefits of retrieval practice while degrading memory for contextual information, including change attributes. Future work may formalize these mechanisms within computational frameworks of key hippocampal subfields that specify how retrieval-driven changes in accessibility and integration evolve over time. Currently, the present results establish some of the core cognitive principles that such models must capture.

## Data Availability

The materials and data are available on the Open Science Framework (https://osf.io/euqmj/).

## Code availability

The analysis code is available on the Open Science Framework (https://osf.io/euqmj/).
